# Examining Weight Suppression, Leptin Levels, Glucagon-Like Peptide 1 Response, and Reward-Related Constructs in Severity and Maintenance of Bulimic Syndromes: Protocol and Sample Characteristics for a Cross-Sectional and Longitudinal Study

**DOI:** 10.2196/66554

**Published:** 2025-04-08

**Authors:** Pamela K Keel, Lindsay P Bodell, Sarrah I Ali, Austin Starkey, Jenna Trotta, J Woody Luxama, Chloé Halfhide, Naomi G Hill, Jonathan Appelbaum, Diana L Williams

**Affiliations:** 1 Department of Psychology Florida State University Tallahassee, FL United States; 2 Department of Psychology Western University London, ON Canada; 3 Department of Psychology Louisiana State University Baton Rouge, LA United States; 4 College of Medicine University of Central Florida Orlando, FL United States; 5 Precisionary Instruments Ashland, MA United States; 6 Department of Psychology Ohio University Athens, OH United States; 7 College of Medicine Florida State University Tallahassee, FL United States; 8 Kravis Department of Integrated Sciences Claremont McKenna College Claremont, CA United States

**Keywords:** binge eating, weight suppression, leptin, glucagon-like peptide 1, insulin, reward, satiation, longitudinal, behavior, Research Domain Criteria

## Abstract

**Background:**

Bulimia nervosa and related syndromes (BN-S) characterized by binge eating vary considerably in illness severity and course. Using the Research Domain Criteria framework of the National Institute of Mental Health, we developed a model positing that the same set of physiological consequences of weight suppression (WS; defined as the difference between the highest and current adult body weight) contribute to binge-eating severity and maintenance by (1) increasing the drive or motivation to consume food (reward valuation effort [RVE]) and (2) decreasing the ability for food consumption to lead to a state of satiation or satisfaction (reward satiation).

**Objective:**

Our funded project aimed to test concurrent associations among WS, physiological factors (leptin concentrations and postprandial glucagon-like peptide 1 [GLP-1] response), behavioral indicators of RVE (breakpoint on progressive ratio tasks) and reward satiation (ad-lib test meal intake), self-report of these core constructs, and binge-eating severity in BN-S (aim 1); test prospective associations to determine whether WS predicts BN-S maintenance in longitudinal models and whether posited mediators also predict BN-S maintenance (aim 2); and determine whether associations between WS and BN-S severity and maintenance are mediated by alterations in leptin levels, GLP-1 response, RVE, and reward satiation (aim 3).

**Methods:**

We aimed to recruit a sample of 320 women with BN-S or noneating disorder controls, with BMI from 16 kg/m^2^ to 35 kg/m^2^, for our study. The study included diagnostic interviews; questionnaires; height, weight, and percentage of body fat measurements; weight history; fasting leptin level; postprandial GLP-1 and insulin responses to a fixed meal; and ad-lib meal and progressive ratio tasks to behaviorally measure reward satiation and RVE, respectively, at baseline, with at least 78.1% (250/320) of the participants providing data at 6- and 12-month follow-up visits. Data will be analyzed using structural equation models to test posited pathways.

**Results:**

Data collection began in November 2016 and ended in April 2023, pausing in-person data collection from March 2020 to February 2021 due to the COVID-19 pandemic. Of 399 eligible women enrolled, 290 (72.7%) provided clinical, behavioral, and biological data at baseline, and 249 (62.4%) provided follow-up data. Measures demonstrated strong psychometric properties.

**Conclusions:**

We seek to identify biobehavioral predictors to inform treatments that target key factors influencing the severity and course of binge eating. These data, supported solely through federal funding, can inform questions emerging from recent interest and controversy surrounding the use of GLP-1 agonists for binge eating.

**International Registered Report Identifier (IRRID):**

RR1-10.2196/66554

## Introduction

### Background

On March 6, 2013, the National Institute of Mental Health (NIMH) released a request for applications titled “Advancing Eating Disorders Research through Dimensional Studies of Biology and Behavior (R01)” to stimulate research using the Research Domain Criteria (RDoC) framework to identify mechanisms underlying eating disorders. In response, our team submitted an application that addressed key requirements. Specifically, we proposed a model that (1) was transdiagnostic, bridging categories in the *Diagnostic and Statistical Manual of Mental Disorders, Fifth Edition* (*DSM-5*) that involve recurrent binge eating; (2) was based on 2 RDoC positive valence domain constructs to explain the 2 defining features of binge eating in the *DSM-5*, including overconsumption of food and loss of control (LOC) over eating; (3) measured constructs and clinical features dimensionally, from a state of health to disease; and (4) used 3 units of analysis, including self-report via interview and questionnaire and biological and behavioral measurement. Our model translated work emerging from basic neuroscience studies of ingestive behavior in rodents to understand the severity and maintenance of bulimia nervosa (BN) and related syndromes (BN-S). This report provides an overview of our explanatory model, supporting literature informing our aims and hypotheses; our protocol for testing hypotheses and adjustments required by the onset of the COVID-19 pandemic; our data analytic plan; our timeline, including recruitment and retention over follow-up; a description of our sample; and psychometric properties of our measures. With data collection completed, this report addresses the feasibility of our approach to testing our model, and our discussion focuses on the implications of potential findings for the assessment, diagnosis, and treatment of eating disorders characterized by binge eating.

### Explanatory Model

A comprehensive review of our explanatory model, including background literature and preliminary data, is available in an open-access article [1]. We proposed that weight suppression (WS), originally defined as the difference between the lifetime highest adult weight and current weight [2], represented a dimensional, transdiagnostic risk and maintenance factor for binge eating in eating disorders. Furthermore, we proposed that WS, independent of current adiposity, contributed to reduced circulating leptin levels, which contributed to a blunted glucagon-like peptide 1 (GLP-1) response to food intake, and that these reductions contributed to alternations in 2 RDoC constructs, reward valuation and reward satiation. Reward valuation represents “benefits of a prospective outcome [...] by reference to external information, social context (eg, group input), and prior experience” [3], and reward valuation effort (RVE) can be measured as the amount of work a reward is worth [4]. In contrast, reward satiation represents “the change in incentive value of a reinforcer over time as that reinforcer is consumed or experienced” [3], and reward satiation can be measured as the termination of reward consumption when it is freely available [4]. Disrupted RVE explains excessive appetitive drive to consume a reward, despite its costs, leading to LOC while eating. Perturbed reward satiation explains the diminished ability to reach a state of completion or satisfaction during consumption, leading to excessive food intake. LOC and excessive food intake represent the defining features of binge-eating episodes, and our model sought to explain both the severity and maintenance of BN-S defined by binge-eating episodes across *DSM-5* eating disorder diagnoses.

### Supporting Literature

Women with BN often have a BMI within the range considered healthy [5], belying an average WS of 7.8 kg [6-14]. Cross-sectional and prospective studies support significant associations between greater WS and BN-S severity [9,14,15] and maintenance [6,8,9], controlling for age, BMI, body image disturbance, and dietary restraint [9,14]. In addition, robust evidence supports that greater WS predicts future weight gain [7,10,16-19]. This last point underscores how WS represents a state resulting from weight loss, which is difficult to maintain due to biological consequences known to influence ingestive behaviors. Our literature review focused on findings translated from basic neuroscience into clinical research that contribute to weight gain and may also impact the risk for binge-eating episodes.

### Biological Consequences of Weight Loss

Weight loss includes loss of white adipose tissue, the primary source of the hormone leptin [20]. Leptin crosses the blood-brain barrier, providing a signal of stored energy to neural regions influencing ingestive behavior [20]. In addition to significant and large (*r*>0.90) positive associations between fat mass and circulating leptin levels [21], weight loss impacts leptin levels independently of BMI [21,22]. Our previous work supported a significant association between greater WS and lower leptin levels, controlling for BMI [23,24] and body fat percentage [23].

Importantly, although WS and leptin levels represent states rather than traits, both demonstrate relative stability throughout the day and are unlikely to directly influence eating onset and termination. Instead, leptin modulates peripherally released meal-related signals that dynamically respond to changes in nutritional intake and impact ingestive behavior [25]. These signals include ghrelin, cholecystokinin, insulin, glucose-dependent insulinotropic polypeptide, secretin, peptide tyrosine tyrosine, insulin-like peptide 5, neurotensin, substance P, and GLP-1. The hormones relay information to the brain about acute changes in energy needs via stimulation of the vagus nerve, with some signals crossing the blood-brain barrier to bind to receptors in neural circuits that impact feeding [25]. Among these, our model focused on GLP-1 based on emerging research on its role in both reward valuation and reward satiation led by one of our investigators [26-30].

GLP-1 is released by L cells in the intestine, and research in rats suggested that leptin potently stimulated postprandial GLP-1 release via leptin receptors on intestinal L cells [31,32]. Thus, our model posits that lower leptin level is associated with blunted postprandial GLP-1 response. Supporting this prediction, individuals with higher leptin levels have demonstrated more robust GLP-1 responses to food intake [33]. These cross-sectional associations appear to reflect the influence of leptin on GLP-1 levels, rather than the reverse, as neither meal-induced increases in GLP-1 nor exogenous GLP-1 administration influence leptin levels in healthy volunteers [34,35]. Thus, our model predicts associations among greater WS, lower leptin levels, and blunted GLP-1 response to food intake, which then influence food intake via alternations in RVE and reward satiation.

### Impact of Peripheral Leptin Levels and GLP-1 Response on Reward Valuation and Reward Satiation

Circulating leptin crosses the blood-brain barrier and binds to leptin receptors in many brain regions, including those involved in reward valuation (ventral tegmental area [VTA] and nucleus accumbens [NAc] [36] and reward satiation (several regions of the hypothalamus and the hindbrain) [37]. Peripherally administered leptin inhibits dopamine projections from the VTA to the NAc [36]. In the arcuate nucleus (Arc) of the hypothalamus, leptin inhibits neurons containing neuropeptide Y and agouti-related protein (NPY/AgRP) and activates neurons containing pro-opiomelanocortin and cocaine- and amphetamine-regulated transcript (CART) [37]. Mesolimbic dopamine signaling impacts many aspects of reward responsiveness [4,38], including RVE, measured as greater effort (breakpoint) in a progressive ratio (PR) task for reinforcers (eg, food, drugs of abuse, and intracranial self-stimulation) [39-42]. Manipulation of leptin receptor function in the VTA supports leptin’s role in reward valuation. In a PR task for food reward, breakpoint was increased by knockdown of leptin receptor expression in the midbrain, including the VTA, but not the hypothalamus [43] and directly in the VTA, but not the substantia nigra [44].

Leptin activation of pro-opiomelanocortin/CART neurons decreases food intake in ad-lib meals across species, including humans [45]. In contrast, NPY/AgRP potently stimulate increased food intake [37]. Thus, when an organism loses adipose tissue (a state marked by WS), leptin levels decrease, activation of pro-opiomelanocortin/CART neurons decreases, and NPY/AgRP neurons remain active and increase ad-lib intake to return the organism to a state of energy balance [37]. Leptin infusions in the Arc [46] decrease ad-lib food intake in rats, and selective deletion of leptin receptors in pro-opiomelanocortin and AgRP neurons increased meal size in mice [47]. Thus, lower leptin levels contribute to diminished responsiveness to satiating signals during food intake. However, the clear causal effects of acute leptin administration in animal models may not reflect the physiological role of leptin in humans, given the noted within-day stability of both WS and circulating leptin levels. That is, an organism cannot lose and gain sufficient white adipose tissue from one meal to the next or within one meal to account for meal initiation or termination via physiological changes in leptin levels. Moreover, our previous work supported significant associations between greater WS and both lower leptin levels and a higher breakpoint on a PR task, and it also found a small negative association between leptin levels and breakpoint [23], further supporting a potential intermediary role for GLP-1.

Peripheral GLP-1 stimulates the vagus nerve, causing activation of the nucleus of the solitary tract (NTS), where central GLP-1 preproglucagon neurons are located [48]. Similar to the effect of leptin levels on GLP-1 release in the periphery, central leptin administration enhances GLP-1 release from preproglucagon neurons of the NTS [49,50]. GLP-1 neurons of the NTS project to multiple brain regions, including the VTA and NAc, where GLP-1 influences RVE [26,31,50-52], as well as to the Arc, paraventricular nucleus of the hypothalamus, and hindbrain, where it contributes to reward satiation [31,52]. In rats, 50% of VTA dopamine neurons express GLP-1 receptors [51], 30% of GLP-1 neurons in the NTS project to the VTA [53], and 40% project to the NAc [26,53]. This positions GLP-1 as a prime candidate for examining how *acute* changes in food intake influence reward pathways in the brain. Infusion of the potent GLP-1 agonist Exendin 4 in the VTA and NAc reduced breakpoint on a food PR task [54], and peripheral administration of a GLP-1 receptor antagonist blunted gastrointestinal nutrient-induced suppression of breakpoint on a food PR task [28]. Exendin 4 diminished conditioned place preference for cocaine [55], implicating GLP-1 signaling in reward responsiveness to a nonfood, noncaloric reinforcer. In healthy humans, meal-induced increases in GLP-1 levels reduced willingness to work for food rewards [56]. Thus, similar to leptin, GLP-1 appears to reduce RVE; however, work in humans has not clearly dissociated the effect of GLP-1 on reward valuation from its clear impact on reward satiation.

Similar to leptin, GLP-1 agonist treatment activates pro-opiomelanocortin and CART neurons [57], and peripheral or central GLP-1 administration suppresses ad-lib food intake and meal size in rodents [58-60]. In humans, peripheral GLP-1 infusion increased satiation and decreased food intake [61], and a meal pattern that increased GLP-1 response was associated with a 10% reduction in food intake during a subsequent ad-lib meal [56]. Moreover, since we initiated data collection to test our model, a plethora of research has emerged demonstrating the effects of GLP-1 agonists on weight via changes in food intake [25,62], and peripherally administered GLP-1 agonists cross the blood-brain barrier where they exert central effects on ingestive behaviors [63].

To summarize, animal-based studies show that leptin and GLP-1 reduce RVE through inhibitory effects in the mesolimbic dopamine pathway and increase reward satiation through a combination of inhibitory and excitatory actions in the Arc. We predicted that lower leptin levels would contribute to blunted postprandial GLP-1 release, which in turn would contribute to both increased RVE and decreased reward satiation. These behavioral consequences increase weight gain and increase the risk of experiencing large, out-of-control binge-eating episodes. We predicted that increased reward valuation would be associated with, and predict, increased frequency of LOC over eating, while decreased reward satiation would be associated with, and predict, consuming unusually large amounts of food.

Although no previous study has examined posited associations dimensionally and prospectively in humans, eating disorders characterized by binge-eating episodes are associated with lower leptin levels [23,64]; reduced GLP-1 response [65,66]; increased RVE, as measured by breakpoint on PR tasks [23,67-70]; and decreased satiation via greater food intake in ad-lib meals [71-77] compared to controls. Our laboratory was able to demonstrate hypothesized differences between women with BN and noneating disorder controls on each of these factors [23,66,78] and extended evidence of blunted GLP-1 response [66] and decreased satiation [78] in women with BN compared to those with purging disorder, which is a condition characterized by purging in the absence of binge-eating episodes [5]. This last finding addresses model specificity to eating disorders characterized by binge eating defined by LOC while eating an unusually large amount of food [5]. Finally, our cross-sectional analyses found that leptin levels statistically mediated the association between WS and reported duration of illness in BN-S [24].

### Aims

Our funded project included the following aims. Aim 1 was to test concurrent associations among WS, physiological factors (leptin concentrations and postprandial GLP-1 response), behavioral indicators of RV-E (breakpoint on PR tasks) and reward satiation (ad-lib test meal intake), self-report of these core constructs, and binge-eating severity in BN-S. Aim 2 was to test prospective associations to determine whether WS predicts BN-S maintenance in longitudinal models and whether posited mediators also predict BN-S maintenance. Aim 3 was to determine whether associations between WS and BN-S severity and maintenance are mediated by alterations in leptin levels, GLP-1 response, RVE, and reward satiation.

### Hypotheses

For examining BN-S severity using cross-sectional data, we hypothesized that greater WS would be associated with lower leptin levels, and lower leptin levels would be associated with lower postprandial GLP-1 response. We hypothesized that lower postprandial GLP-1 response would be associated with greater reward valuation and lower reward satiation. We predicted that greater reward valuation would be associated with a higher frequency of LOC over eating. We predicted that lower reward satiation would be associated with larger eating episode sizes, extending from eating episodes that were not large in control participants to objectively large binge-eating episodes in participants with BN-S across the full severity range (“eating/binge-eating episode size”). We also predicted a significant indirect pathway from WS to LOC frequency via leptin levels, GLP-1 response, and reward valuation. We predicted a significant indirect pathway from WS to eating/binge-eating episode size via leptin levels, GLP-1 response, and reward satiation. If supported, findings would demonstrate that biological concomitants of WS explain differences in the severity of binge eating via alterations in reward valuation and reward satiation.

For examining BN-S maintenance using longitudinal data, we hypothesized prospective associations in which greater WS would prospectively predict lower leptin levels, which would be associated with lower postprandial GLP-1 response. We hypothesized that lower postprandial GLP-1 response would be associated with greater reward valuation and lower reward satiation. We predicted that greater reward valuation would prospectively predict a higher frequency of LOC over eating. We also predicted that lower reward satiation would prospectively predict larger eating episode sizes.

To examine the mediation of the association between WS and BN-S severity and maintenance via biobehavioral alterations, we predicted that reward valuation and reward satiation would mediate associations between WS and changes in LOC frequency and eating/binge-eating episode size. If supported, findings would translate findings from animal models in neuroscience to clinical outcomes in humans and identify GLP-1 as a potential target in future treatment studies of eating disorders characterized by binge eating, including BN-S.

## Methods

### Protocol

**Figure 1:**
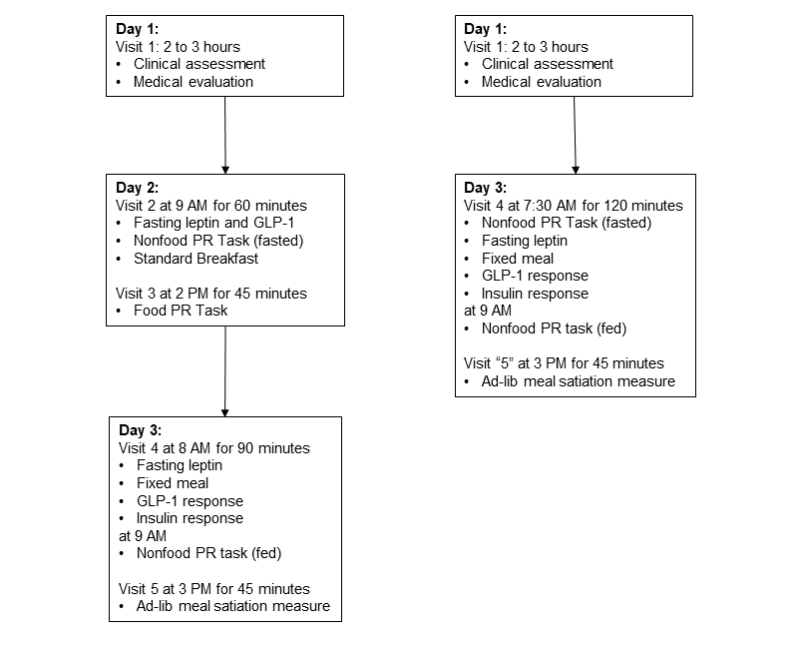
Study protocol for participants enrolled before (left) and after (right) the onset of the COVID-19 pandemic. GLP-1: glucagon-like peptide 1; PR: progressive ratio.

All data and sample collections, as well as assays, were conducted in a clinical research laboratory at Florida State University (FSU). Figure 1 presents an overview of study visits for participants enrolled before and after the onset of the COVID-19 pandemic.

#### Day 1 or Visit 1: Psychological and Medical Evaluation

At baseline, all participants completed in-person structured clinical interviews with clinical doctoral students and questionnaires on a laboratory computer (Measures section); a pregnancy test; an objective assessment of height, weight, percentage of body fat, and vital signs; and a medical screening. At baseline, participants also played Angry Birds (Rovio Entertainment) for 2 minutes, tasted M&M’s (Mars, Inc) and frozen yogurt, and consumed the fixed meal used in visit 4 to ensure the ability to follow instructions in subsequent visits. After the onset of the COVID-19 pandemic, remote follow-up clinical assessments were offered via a Zoom (Zoom Communications) link, which complies with the Health Insurance Portability and Accountability Act (HIPAA), for interviews and a secure web-based link for questionnaires.

#### Days 2 and 3 Visits

The original protocol included 2 visits on day 2 at 9 AM (visit 2) and 2 PM (visit 3) and day 3 at 8 AM (visit 4) and 3 PM (visit 5). Start times were adjusted by up to 1 hour before or after indicated times, and any adjustment was held constant across a participant’s visit. Participants were asked to abstain from eating or drinking anything except water and from purging or exercising after 11 PM before and between visits on both days. Participants were required to leave personal belongings, including mobile phones, books, and other items, in a bin outside the testing rooms. At the beginning of the morning visits, height, weight, body fat percentage, and vital signs were objectively measured, and a screening confirmed compliance with instructions and captured the past week’s eating patterns and eating disorder symptoms. Task instructions were presented in print and via audio recording. We used digital video monitoring to detect technical problems and participant noncompliance during behavioral tasks. The revised protocol eliminated the day 2 visit but retained visit 2 tasks (Visit 2: Leptin, GLP-1, RVE for a Nonfood Reinforcer in Fasted State state).

#### Visit 2: Leptin, GLP-1, and RVE for a Nonfood Reinforcer in Fasted State

Participants had 5 ml of blood drawn by a registered nurse and completed momentary assessments before and after a PR task to play Angry Birds [79]. Briefly, participants were instructed that they could earn 1 minute of playtime in Angry Birds by pressing the space bar; the task consisted of up to 10 trials for up to 10 minutes of playtime, with the number of required presses increasing across trials (50 presses for trial 1, 250 presses for trial 2, 450 presses for trial 3, and so on up to 1850 presses for trial 10). Participants were instructed that they should continue for as long as they wanted to play the game; they could stop at any time, and there were no right or wrong answers. When participants reached the criterion for a trial, a window opened, and they could play the game for 1 minute. After 1 minute, the screen automatically closed, and the participants were returned to the PR task, where they could work to gain access to continue the game. RVE was operationalized as a breakpoint, defined as the number of key presses in the last completed trial [80]. At 10 AM, participants consumed a standardized breakfast (300 kcal of yogurt parfait and juice) [23].

#### Visit 3: RVE for a Food Reinforcer

Participants completed momentary ratings before and after a PR task for M&M’s [23], using the same design, instructions, and PR schedule as the nonfood task, to earn 10 M&M’s per trial, with the potential to earn up to 100 M&M’s over 10 trials. When participants reached the criterion for a trial, 10 M&M’s dropped into a cup, and they were instructed to consume all M&M’s before continuing to work for additional M&M’s. The revised protocol eliminated the food PR task and associated momentary ratings.

#### Visit 4: GLP-1 Response to a Fixed Meal and RVE for a Nonfood Reinforcer in Fed State

A registered nurse placed an indwelling catheter into the participant’s arm and allowed the participant to rest for 5 minutes. Participants completed momentary ratings before the nurse drew a fasting blood sample (5 ml) at –5 minutes for leptin, GLP-1, and insulin. Then, participants consumed a fixed liquid meal (Ensure Plus, Abbott Nutrition; 900 kcal in 660 g of fluid: 30% fat, 15% protein, and 55% carbohydrate) from –5 to 0 minutes. To capture GLP-1 and insulin responses to the fixed meal, 2 ml blood samples were drawn at +5, +15, and +30 minutes. Momentary ratings were completed immediately before each blood draw. After the last blood draw, participants had their intravenous catheter removed, rested for 5 minutes, and completed momentary ratings before and after the nonfood PR task (fed state). In the revised protocol, the fasted nonfood PR task and associated momentary ratings were completed before the first fasting blood draw, such that blood samples were drawn shortly after, rather than before, the fasted nonfood PR task.

Breakpoint on the nonfood PR task demonstrated excellent test-retest reliability (intraclass correlation [ICC]=0.91; 95% CI 0.80-0.96) over 2 weeks and convergent validity with the food PR task (*r*=0.51; *P<*.001) [79]. Consistent with animal models, breakpoint was lower in fed compared to fasted states across tasks (B=321.01, SE 552.40; *P<*.001). Finally, the nonfood task demonstrated discriminant validity from the measurement of satiation [79].

#### Visit 5: Ad-Lib Meal Assessment of Reward Satiation

Participants completed momentary ratings immediately before and after an ad-lib meal comprising 1.5 quarts (1420 g) of vanilla frozen yogurt (1.5 kcal/g) served at an individual place setting. Participants were presented with instructions in print and audio recorded to eat until they felt full or satisfied. Yogurt was weighed twice both before and after the meal using a top-loading, self-calibrated electronic balance, and the total intake was calculated in grams and kilocalories [81].

#### Training and Interrater Reliability

PKK provided all training and supervision for structured clinical interviewers and held biweekly assessment meetings. Interrater reliability (IRR) was examined via independent coding of audio recordings from 127 (16.5%) out of 769 interviews, randomly selected from each interviewer’s assessments annually.

### Participants

Textbox 1 presents the inclusion and exclusion criteria. Local recruitment was conducted via advertisements on social media platforms (eg, Instagram; Meta Platforms, Inc), billboards, pamphlets distributed to clinics, posters, outreach to college-based student organizations for ethnic or racial minority groups and churches, and emails to students at local universities with a link to a web-based eligibility screening. We received permission to send the mass email to female students aged 18 to 35 years once per academic term for all terms in which we recruited new participants at FSU and for one of the terms at a local Historically Black College and University. The mass email was the most effective recruitment tool, yielding the largest response. All prospective participants underwent telephone screenings to assess potential eligibility. Full eligibility was determined during the in-person assessment at baseline. The NIMH Data Archive (NDA) collection includes data from all eligible participants (N=399) and a small number of participants (n=6, 1.5%) who were later determined not to meet full eligibility criteria (details present in the Data Availability section).

**Eligibility criteria for participants.**

**Inclusion criteria**

- Sex: female
- Age: 18 to 35 years
- BMI: 16 to 35 kg/m^2^
- Free of psychotropic medications or stable dose of selective serotonin reuptake inhibitors for 8 weeks
- Free of alcohol, illicit drugs, and medications for 72 hours before reward valuation effort, reward satiation, and fixed meal tasks

**Additional inclusion criteria for bulimia nervosa and related syndromes**

- Diagnostic and Statistical Manual of Mental Disorders, Fifth Edition (*DSM-5*), binge episodes
  ≥1,000 kcal within a 2-hour period and larger than what most people would eat in a similar context
  Loss of control over eating during the episode
- More than 12 episodes of behavioral eating disorder symptoms (including binge, purging, and nonpurging behaviors) over the past 12 weeks
- Undue influence of weight or shape on self-evaluation, intense fear of gaining weight or becoming fat, or marked distress regarding binge eating
- *DSM-5* criteria for anorexia nervosa binge-purging, bulimia nervosa, binge eating disorder, or other specified feeding or eating disorder using the past 12 weeks as a measure of the past 3 months
- For current other specified feeding or eating disorder, score >16 on the Clinical Impairment Assessment; marked distress regarding binge eating; or impairment in one or more areas of life on the Structured Clinical Interview for DSM-5
- For atypical anorexia nervosa, BMI ≥18.5 to <19 kg/^2^ or >5% BMI reduction over a 1-month period

**Exclusion criteria**

- Medical condition or other treatment that could influence appetite, weight, or ability to participate
- Currently pregnant, nursing, or planning to become pregnant within the next year
- Planning to move >2 hours away from the laboratory within the next year
- Current blood, injection, or injury phobia

**Additional exclusion criteria for noneating disorder controls**

- Current eating disorder symptoms on the Eating Disorder Examination
- Current dietary restriction to lose weight on the Eating Disorder Examination (dietary restriction to prevent weight gain was permitted)
- History of any eating disorder symptoms on the Structured Clinical Interview for *DSM-5*
- Three-Factor Eating Questionnaire cognitive restraint subscale score ≥10
- Clinical Impairment Assessment score ≥16

To measure the severity of BN-S dimensionally from a state of health to disease, we included noneating disorder controls and participants with BN-S ranging from mild to severe. We deliberately recruited more participants with BN-S than controls to collect data appropriate for parametric analyses by minimizing variables with a modal value of 0. This strategy also prioritized the recruitment of participants with BN-S at baseline, as predictors of illness maintenance and the examination of GLP-1 dysfunction as a potential treatment target were most relevant for this group. Finally, this strategy reflected our previous research experience and the expectation that controls would demonstrate limited variance at baseline, when they were required to be free of eating pathology, and over the course of follow-up when the incidence of eating disorders would be rare. With reduced variability, a smaller number was expected to provide sufficiently narrow CIs on any secondary group-based analyses comparing controls to participants with BN-S.

Participants provided multiple and preferred methods of contact (phone calls, SMS text messages, and email). Participants also provided the name and contact information for at least 1 individual who would know how to reach them if the participant could not be contacted by their mobile number and gave permission for us to contact that individual if we lost contact. We used several strategies to enhance retention. First, we offered financial incentives to participants prorated by visit and a bonus for completing all visits as scheduled (details present in the Ethical Considerations section). Second, we provided written and verbal reminders and instructions for subsequent visits via calls, SMS text messages, and emails for 3 days and 1 day before visits and wake-up calls for morning visits. Third, we established clear participation policies: participants who missed 2 scheduled visits without prior notification were discontinued, and we limited rescheduled visits to 3 per sequence. Fourth, if ≥12 weeks passed between the day 1 interview and the final visit, we required confirmation of continued eligibility via reassessment. Finally, a 6-month follow-up could begin 4 to 8 months after baseline, a 12-month follow-up could begin ≥10 months after baseline, and 6-month participation was not required to complete a 12-month follow-up. Assessment dates are included in the NDA collection.

### Measures

#### BMI, WS, and Body Fat Percentage

Current weight and height were measured each day using a digital scale and stadiometer to calculate BMI (kg/m^2^). We observed stability in BMI across days (*r*>0.95; *P*<.001), with no significant changes between days 1 and 2 (Cohen *d*=0.09), 2 and 3 (Cohen *d*=–0.07), and 1 and 3 (Cohen *d*=–0.007). WS was calculated as a percentage of BMI loss from the highest previous BMI, using self-reported highest adult weight at current height to calculate the highest BMI. At 6- and 12-month follow-up visits, we again asked about the highest adult weight to capture possible changes over time. Body fat percentage was measured using bioelectrical impedance analysis (Tanita Corporation of America), which demonstrates high correlations (*r*=0.88 to 0.94) with dual x-ray absorptiometry scans [82], supporting its feasibility (lower cost) and safety (no radiation exposure) in longitudinal studies with larger samples. We measured duration at current weight, time since highest weight, and duration of highest weight via self-report. WS represents our exposure variable, and BMI and body fat percentage represent covariates.

#### Interviews

The *Eating Disorder Examination* (EDE) *17.0D* [83] was selected due to previous evidence of good discriminant validity [84-87], IRR (0.83 to 0.99) [88,89], and good internal consistency of the restraint and body image subscales (Cronbach α>0.70) [90]. The EDE provided 3D outcome variables of eating disorder severity and maintenance, each with high IRR in the current project determined through ICC: LOC frequency (ICC>0.99), size of largest eating/binge-eating episode (in kcal; ICC=0.82), and the Global Scale score (ICC>0.99). LOC frequency represented the total number of eating episodes during the previous 12 weeks during which participants did not feel in control of their eating, regardless of the amount of food consumed. The size of the largest eating/binge-eating episode was captured by asking participants to report the largest amount of food they had eaten during the previous 12 weeks and recording the types and amounts of food consumed within 2 hours and converting these to kilocalories as described previously [78,91]. The EDE also provided scores with high IRR for restraint (ICC>0.99); eating concern (ICC=0.98); weight (ICC=0.99) and shape concerns (ICC>0.99); and symptoms for algorithms to diagnose current *DSM-5* anorexia nervosa (AN), BN, and binge-eating disorder (BED) in combination with objectively measured BMI. EDE symptom frequencies and durations permitted differential diagnoses of other specified feeding or eating disorder (OSFED) proposed by Keel [92]. IRR for symptom frequency or severity (ICC) and associated diagnostic thresholds over 3 months (κ) were good: objective binge episode size (κ=0.71), objective binge episode and inappropriate compensatory behavior frequency (ICC=0.97 and κ=0.93; ICC=0.96 and κ=0.91, respectively), fear of gaining weight or becoming fat (ICC=0.99 and κ=0.91), behavior to prevent weight gain (ICC=0.96 and κ=0.96), weight misperception (ICC=0.98 and κ=0.95), self-evaluation unduly influenced by weight (ICC=0.87) or shape (ICC=0.89 and κ=0.90), characteristics associated with binge episodes (ICC=0.78 and κ=0.73), and marked distress regarding binge eating (ICC=0.91 and κ=0.91). Marked distress regarding binge eating on the EDE was 1 of the 3 indicators of clinical significance for OSFED. The EDE was administered at baseline and 6- and 12-month follow-up assessments.

The Structured Clinical Interview for Diagnostic and Statistical Manual of Mental Disorders, Fifth Edition (SCID-5) [93] captured eating (lifetime) and related disorder diagnoses (lifetime and current). Previous work supported good IRR for SCID diagnoses, with κ=0.92 for major depressive disorder, κ=0.75 for dysthymic disorder, κ=0.81 for any substance use disorder, κ=0.85 for panic disorder, κ=0.91 for social phobia, κ=0.73 for specific phobia, κ=1.00 for obsessive-compulsive disorder, and κ=0.90 for posttraumatic stress disorder [94]. We confirmed the absence of lifetime eating disorder symptoms in noneating disorder controls by ignoring skip rules [95]. The OSFED addendum provided a second indicator of clinical significance. The overview covered current and past treatment, including medication use, to confirm eligibility. At follow-up, we evaluated but did not exclude based on the use, type, dose, and duration of medication, and information may be included as covariates. Follow-up assessments focused on the period since the previous interview (eg, the past 6 months) to reduce participant burden. Current depressive and substance use disorders are covariates.

#### Self-Reported Questionnaires

C*linical Impairment Assessment* [96] established impairment and distress specifically linked to eating disorder symptoms in the domains of personal, social, and cognitive function. Previous work supported high internal consistency (>0.90), test-retest reliability (0.86), and concurrent and discriminant validity [96,97]. A score ≥16 was a third indicator of clinical significance for OSFED [96].

The *Three-Factor Eating Questionnaire* [98] comprises scales for cognitive restraint, which has successfully differentiated dieters from nondieters for our study’s threshold for determining eligibility of controls, and disinhibition and hunger, which distinguish between purging women based on binge episode size [89,91]. Previous work supported good 1-month test-retest reliabilities for cognitive restraint, disinhibition, and hunger subscales of >0.90, 0.80, and 0.83, and internal reliabilities of >0.90, 0.91, and 0.85, respectively [98].

The *Visual Analog Scales* (VAS) assessed momentary states by presenting a single item on each page of a booklet with a 100-mm horizontal line anchored from “none or not at all” to “extreme or extremely.” These provided self-reported levels of reward valuation and reward satiation during behavioral tasks. Participants marked the line to record how they felt “Right Now,” regarding hunger, fullness, satiation, how much they wanted M&M’s and to play the game, how much they liked M&M’s and the game, and how rewarding they found M&M’s and gameplay, urge to binge, urge to vomit, nausea, stomach discomfort, sadness, anxiety, tension, and preoccupation with weight and shape. VAS dimensional scores are sensitive to momentary changes associated with postprandial gut peptide responses to a fixed meal, and responses to an ad-lib and fixed meal differ significantly between controls and participants with eating disorders [78,99].

The following measures were included to supplement the clinical description of participants based on the measures described earlier for determining eligibility, exposure, and outcome variables; in response to comments provided during the grant review (Multimedia Appendix 1); and in anticipation of potential questions that may emerge during the review of manuscripts presenting tests of our model.

The *Body Shape Questionnaire* [84] measured feelings and attitudes about body shape and weight as a possible covariate, based on previous evidence of its high test-retest (0.88) [100], high internal reliability (>0.98) [100,101], and good discriminant and concurrent validity.

The *Positive and Negative Affect Scale* [102] assessed trait levels during visit 1 and state levels of positive and negative affect before and after biobehavioral assessments in visits 2 to 5 to examine these as possible covariates of RVE and reward satiation. The Positive and Negative Affect Scale is a widely used measure due to its high internal consistency, test-retest reliability, and construct validity [102,103].

The *Behavioral Inhibition and Behavioral Activation Scales* [104] and the *Sensitivity to Punishment and Sensitivity to Reward Questionnaire* [105] measure reward-related traits. The former measure has demonstrated high internal consistency (0.82) in previous work [104] and concurrent and discriminant validity with the latter measure in eating disorder samples [105].

### Leptin, GLP-1, and Insulin Assessments

Enzyme-linked immunosorbent assays (MilliporeSigma) of plasma samples were used to measure leptin (EZHL-80SK) [106], active GLP-1 (EZGLPHS-35K [107], total GLP-1 [EZGLP1T-36K] [108]), and insulin (EZHI-14K) [109] levels. Insulin was added to the protocol in response to grant reviewers’ (Multimedia Appendix 1) concerns about the narrow focus on GLP-1 and the evidence that insulin, similar to GLP-1, might influence reward valuation, in addition to its already defined role in satiation [63]. Blood samples were collected into prechilled K2 EDTA vacutainers, following protocol instructions, including the preparation of vacutainers with DPP-IV inhibitor (10 µl/ml of blood) for active GLP-1, before centrifuging at 2000 revolutions per minute for 15 minutes at 4 °C. Plasma was then pipetted into prechilled, aliquot tubes labeled by participant number, analyte, visit, date, and sample number, and immediately placed on ice and transferred to a –80 °C freezer. We extracted up to 4 aliquots, providing up to 3 backup samples for each analyte.

DLW supervised all assays and approved results without knowledge of the participant’s clinical status. All participant samples were run in duplicate in 1 assay (eg, baseline leptin from visits 2 and 4) and batched to ensure a balanced representation of control and BN-S participants across assays. When results were outside the detection limits or the intra-assay coefficient of variation (CV) exceeded 10%, backup aliquots were run in subsequent assays to ensure reliable estimates. Preliminary analyses indicated low stability of fasting active GLP-1 levels from visit 2 to 4 (*r*_191_=0.18, *P=*.01) at baseline, raising concerns about fasting active GLP-1 as a sole measure of individual differences in GLP-1 function across visits. We added assays of total GLP-1 in backup samples to the protocol, which had been proposed in our grant application in the section on potential pitfalls and solutions in response to grant reviewers’ (Multimedia Appendix 1) concerns about measuring active GLP-1. Total GLP-1 demonstrated adequate stability for fasting concentrations from visit 2 to 4 (*r*_193_=0.70; *P<*.001). The NDA collection includes both active and total GLP-1 values. For participants enrolled after the onset of the COVID-19 pandemic, all GLP-1 values represented total GLP-1 and came from visit 4. Importantly, multilevel model analyses demonstrate that visit 4 VAS fullness ratings were significantly predicted by changes in active GLP-1 (B=0.83, 95% CI 0.65-1.01; t_734_=9.06; *P<*.001) and total GLP-1 (B=0.40, 95% CI 0.27-0.54; 2-tailed t_708_=5.91; *P<*.001) values, with greater effect size for active GLP-1 level. Thus, both provide valid indicators of GLP-1 function, with total GLP-1 providing more stable, “trait”-like information and active GLP-1 value providing more sensitive, “state”-like information.

The kit user guides and information published on the MilliporeSigma (Merck Group) website and included in assay kits reported the following upper limits of interassay and intra-assay CVs, respectively: 6.2% and 4.6% for leptin [106], <15% and <10% for active GLP-1 [107], <12% and <5% for total GLP-1 [108], and 11.4% to 6.95% for insulin [109]. In our project, mean intraassay and interassay CVs, respectively, were 4.3% and 9% for leptin, 7.4% and 9.5% for active GLP-1, 2.9% and 10.3% for total GLP-1, and 3.9% and 8.9% for insulin. We observed comparable or better CVs compared to published estimates, except for the higher interassay CV for leptin. Date and assay (numbered consecutively) are included in the NDA collection so that future users can control for interassay variability.

### Data Structure and Missing Data

NDA data are organized by measure and stored in long form. Variable names indicate visits via the “_#” naming convention. Regardless of when participants were enrolled, variable names retained their original designations. The “_2” suffix reflects variables collected before, during, and after the fasted nonfood RVE task. Similarly, data collected from the fixed meal assessment of GLP-1 and insulin responses are identified by the “_4” suffix, and data collected from the ad-lib meal assessment are identified by the “_5” suffix. Each visit has a “Notes” column to indicate protocol deviations that may impact data quality. In addition to attrition, participants skipping questions, experimenter error, and equipment failures contributed to missing values. Technical problems included M&M’s jamming the dispenser and not dispensing the reward after participants reached the criterion (the apparatus was redesigned part way through the project to minimize this problem) and the computer program freezing. When a participant’s effort for reward exceeds the planned ratio, data from the session are included in the data collection but will not be included in planned analyses. Refer to the study by Keel et al [79] for an example of main analyses, which excluded values from sessions with noted problems and sensitivity analyses, including all available data. The NDA collection includes all available data; several variables must be calculated (eg, breakpoint).

### Data Analytic Plan

For concurrent tests of severity, we plan to use structural equation models (SEM) in MPlus (version 8; Muthén & Muthén) to obtain estimates of overall model fit; account for shared variance; and provide path estimates within the model, including tests of indirect effects using bias-corrected bootstrapped CIs with 10,000 samples to include all available data in the model from the 399 eligible participants enrolled in the study. This strategy includes running correlations between all variables in the model as a first step, followed by SEM with WS as the sole exogenous variable and the remaining variables as endogenous variables. Both RVE and reward satiation will be modeled as latent variables, with pathways from both behavioral tasks and self-reported states from VAS ratings. For prospective associations and temporal mediation predicting illness maintenance, we proposed cross-lagged SEM with bootstrapping methods for testing indirect effects [110]. Model fit will be evaluated with common fit indices, with the following thresholds for interpreting good fit: root mean square error of approximation, comparative fit index, and Tucker-Lewis Index ≥0.90; root mean square error of approximation value ≤0.05, 95% CI >0.00 to ≤0.08; and standardized root mean square residual ≤0.08 [111-116]. Coefficients and their 95% CIs will be used to evaluate the significance of hypothesized direct and indirect pathways, and only direct pathways that are in the predicted direction, with 95% CIs that do not include 0, will be interpreted as supporting the a priori hypotheses generated by our model.

Sensitivity analyses will evaluate the impact of adding covariates with pathways to each endogenous variable in the model on overall model fit and parameter estimates. Covariates include age, BMI, body fat percentage, enrollment before and after the onset of the COVID-19 pandemic, hormonal contraceptive use, selective serotonin reuptake inhibitors use, current depressive disorder diagnosis, and current substance use disorder diagnosis. Because sensitivity analyses involve nonnested models, we will evaluate whether there is a qualitative change in the adequacy of model fit, from inadequate to adequate or from adequate to good. When there is no qualitative change in model fit, lower values on the Akaike information criterion and Bayesian information criterion will be interpreted as evidence of improved fit.

### Power Analyses

We conducted power analyses in R (R Foundation for Statistical Computing) with PowMedR for mediation as our least-powered analyses. Across the posited indirect effects (eg, GLP-1 mediates the association between WS and reward satiation), a sample size of n=195 provides 80% power with path coefficients ≥0.22 from the initial variable to the mediator and from the mediator to the outcome variable, while controlling for the initial variable. Analyses on multiple imputed datasets indicated 80% power with path coefficients ≥0.20 to test indirect effects. We aimed for longitudinal data from 250 participants to permit exploratory moderation analyses for potentially weaker effects at higher BMIs, where leptin levels might be high despite high WS and high food consumption [1].

### Ethical Considerations

The FSU Institutional Review Board Human Subjects Committee reviewed and approved the study protocol and materials (HSC 2016.15338/STUDY00000353). Written informed consent was obtained from all study participants before their participation. All data have been deidentified and only a study-generated ID number and a globally unique identifier number in the NDA have been retained. For the original protocol, participants were paid US $75 for day 1, US $50 for day 2, US $100 for day 3, and a US $35 bonus for a total potential compensation of US $780 for baseline and 6- and 12-month follow-up visits. For the revised protocol, participants were paid US $75 for day 1, US $110 for day 3, and a US $15 bonus for a total of US $400 for baseline and 6-month follow-up.

## Results

### Overview

The project was funded as an investigator-initiated R01 in August 2016, and data collection began in November 2016. Data collection from participants was completed in April 2023, and assays of analytes were completed in June 2023. Information presented on recruitment and retention addresses the feasibility of our design and provides context on information reported on the reliability of measures in the current sample.

### Timeline and Recruitment

We aimed to recruit 320 women over a 5-year period to provide clinical, biological, and behavioral data. We estimated that 250 (78.1%) out of 320 women would complete 6- and 12-month follow-up visits, including approximately 78% (200/256) with BN-S and 78% (50/64) control participants. By March 2020, we had recruited 301 eligible participants. Due to the COVID-19 pandemic, the laboratory closed that month for in-person assessments. To retain participants with baseline data who had not completed 12-month follow-up, institutional review board (IRB)–approved, NIMH-approved, and HIPAA-compliant remote clinical assessments were added to our protocol.

On February 19, 2021, we reopened the laboratory for in-person follow-up visits for those who had completed all baseline visits before laboratory closure, implementing IRB-approved safety precautions. The precautions included (1) a screening checklist for symptoms, potential COVID-19 exposure, and a temperature check completed with the participant outside the laboratory before admitting them into the laboratory; (2) allowing only 1 participant into the laboratory at a time; (3) reducing study staff to the minimum required to complete a visit; (4) following masking and social distancing guidelines; (5) sanitizing all surfaces before and after running each participant; and (6) continuing to allow participants to complete follow-up clinical assessments via remote means. The final precaution reflected evidence of minimal differences between in-person versus remote follow-up assessments conducted among those enrolled before the onset of the COVID-19 pandemic [117]. We adjusted pandemic-related precautions as needed, including periodically pausing in-person assessments in response to spikes in new variants and relaxing precautions with growing community immunity.

On October 8, 2021, we enrolled our first new participant since laboratory closure, supported by 2 no-cost extensions of our NIMH-funded grant and a US Department of Education Higher Education Emergency Relief Fund awarded to FSU. Before enrolling new participants, and with prior approval from the FSU IRB and the NIMH, we revised the protocol to reduce the number of in-person visits to conserve remaining funds and further minimize the risk of COVID-19 exposures. Changes were also informed by evidence of the reliability and the convergent and discriminant validity of our task for measuring RVE for a nonfood reward [79]. We completed data collection in April 2023. We experienced no known incidents of COVID-19 exposure in our laboratory during the project.

### Retention

**Figure 2:**
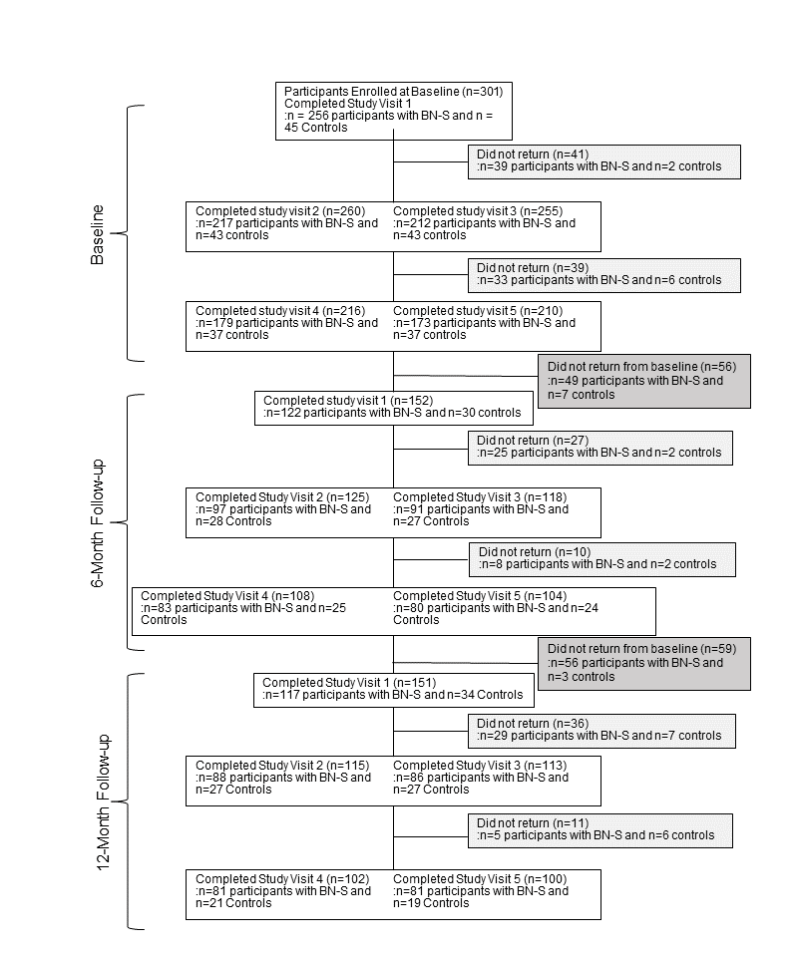
Participant flow through the study for participants recruited before the onset of the COVID-19 pandemic. At the 6-month follow-up, 2 additional participants with BN-S completed questionnaire assessments but did not complete interviews for study visit 1. Participants who completed all visits at baseline were recruited to participate at the 12-month follow-up, whether or not they had completed 6-month follow-up. BN-S: bulimia nervosa and related syndromes.

**Figure 3:**
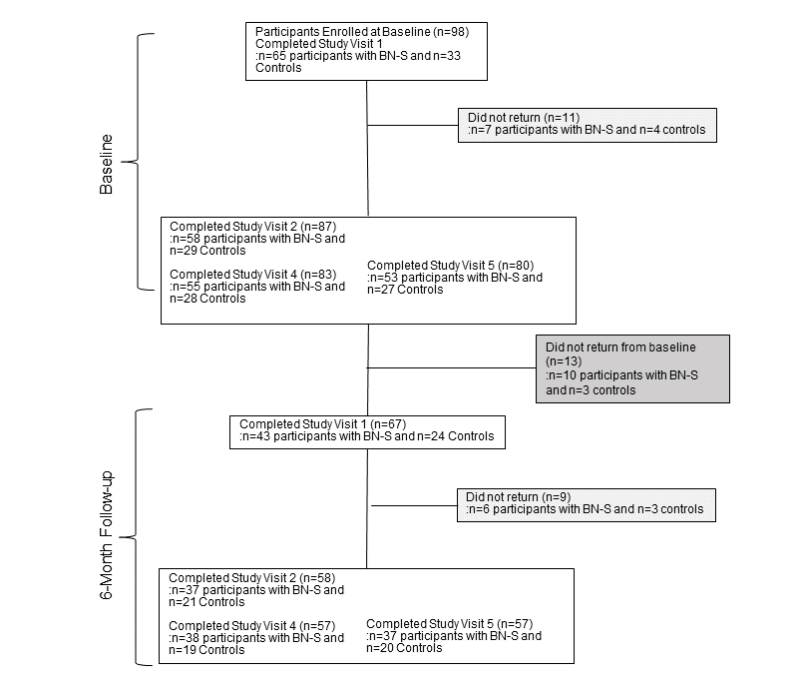
Participant flow through the study for participants recruited after the onset of the COVID-19 pandemic. BN-S: bulimia nervosa and related syndromes.

Figures 2 and 3 show retention across study visits and follow-up for participants enrolled before and after the onset of the COVID-19 pandemic, respectively. Among those 399 eligible, 321 (80.5%) had a current BN-S, 78 (19.5%) were noneating disorder controls. A total of 290 (72.7%) out of 399 women completed all baseline assessments, including 226 (70.4%) out of 321 with BN-S and 64 (82.1%) out of 78 controls (*χ*^2^_1_=4.3; *P=*.04; φ=0.10). Given the uncertainty surrounding the duration of the COVID-19 pandemic–related closures and concerns for participant safety, participants (91/399, 22.8%) without complete baseline assessments were considered ineligible to continue (n=83, 91% with BN-S and n=8, 9% controls). We estimate that the COVID-19 pandemic impacted 3.3% (13/399) participants’ ability to complete baseline assessments, all of whom had BN-S. The mean time between days 1 and 2 was 17.7 (SD 15.1) days, between days 2 and 3 was 16.5 (SD 17.3) days, and between days 1 and 3 was 28.1 (SD 19.3) days. There was no significant difference between groups in days between study visits (*P* values ranged from .72 to .07, and effect sizes were small; Cohen *d*=–0.09 to 0.29).

Among the 290 women eligible for follow-up, 249 (85.9%) provided follow-up data, including 96 (33.1%) with 6-month, 123 (42.4%) with 6- and 12-month, and 30 (10.3%) with 12-month data. Groups did not differ in participation at 6-month (*χ*^2^_1_=3.5; *P=*.06; φ=0.11) or 12-month follow-up (*χ*^2^_1_=0.0; *P=*.95; φ==0.004). The mean time between baseline to 6-month follow-up was 6.0 (SD 2.2) months, 6- to 12-month follow-up was 8.5 (SD 7.8) months, and baseline to 12-month follow-up was 14.6 (SD 5.6) months and did not differ significantly between groups (*P* values ranged from .82 to .30, and effect sizes were small; Cohen *d*=–0.09 to 0.29).

### Sample Descriptors

Table 1 describes sample characteristics. Biological sex as female at birth was required for all participants (Textbox 1), and almost all endorsed being cisgender individuals. We did not assess sexual orientation. According to 2020 US Census data [118], the racial or ethnic composition of Leon County, Florida, was 0.4% American Indian or Alaska Native, 3.8% Asian, 32.1% Black or African American, 8.3% Hispanic or Latino, 0.1% Native Hawaiian or other Pacific Islander, 61% White (54.1% non-Hispanic and non-Latino), and 2.6% multiracial. Racial or ethnic composition of the sample differed from the county population (*χ*^2^_5_=91.9; *P<*.001; Cramer V=0.008), with lower participation among individuals identifying as Black or African American (*χ*^2^_1_=72.2; *P<*.001; φ=0.02) and greater participation among those identifying as Native Hawaiian or other Pacific Islander (*χ*^2^_1_=4.3; *P=*.04; φ=0.004) or multiracial (*χ*^2^_1_=6.7; *P=*.01; φ=0.005) relative to those identifying as White individuals. Furthermore, we observed greater participation among individuals identifying as Hispanic or Latinx compared to non-Hispanic or Latinx (*χ*^2^_1_=193.8; *P<*.001; φ=0.03).

There was no significant association between racial or ethnic identity and whether or not participants completed baseline visits (*χ*^2^_5_=2.1; *P=*.84 and *χ*^2^_1_=0.0; *P=*.99) and 6-month (*χ*^2^_5_=0.6; *P=*.99 and *χ*^2^_1_=1.3; *P=*.72) or 12-month (*χ*^2^_5_=1.6; *P=*.90 and *χ*^2^_1_=0.1; *P=*.79) follow-up (all effect sizes for all associations; η^2^<0.01). Table 2 presents 6-month sample characteristics, and Table 3 presents 12-month sample characteristics.

**Table 1:** Characteristics of the sample at baseline for all eligible participants (N=399).

Characteristic			Total, n (%)		Bulimic syndrome group (n=321), n (%)		Control group (n=78), n (%)
Female			399 (100)		321 (100)		78 (100)
**Gender**							
	Woman	397 (99.5)		320 (99.7)		77 (98.7)	
	Nonbinary	1 (0.2)		0 (0)		1 (1.3)	
	Not reported	1 (0.2)		1 (0.3)		0 (0)	
**Ethnicity**							
	Hispanic or Latino	110 (27.6)		91 (28.3)		19 (24.4)	
	Not Hispanic or Latino	289 (72.4)		230 (71.7)		59 (75.6)	
**Race**							
	American Indian or Alaska Native	2 (0.5)		2 (0.6)		0 (0)	
	Asian	14 (3.5)		8 (2.5)		6 (7.7)	
	Black	47 (11.8)		36 (11.2)		11 (14.1)	
	Hawaiian or other Pacific Islander	2 (0.5)		2 (0.6)		0 (0)	
	Multiracial	23 (5.8)		20 (6.2)		3 (3.8)	
	White	311 (77.9)		253 (78.8)		58 (74.4)	
**Educational level**							
	Part college	346 (86.7)		284 (88.5)		62 (79.5)	
	Associate degree	10 (2.5)		6 (1.9)		4 (5.1)	
	Bachelor degree	10 (2.5)		6 (1.9)		4 (5.1)	
	Part graduate school	30 (7.5)		24 (7.5)		6 (7.7)	
	Graduate degree	3 (0.8)		1 (0.3)		2 (2.6)	
**Relationship status**							
	Married or having a partner	33 (8.3)		25 (7.8)		8 (10.2)	
	Divorced or annulled	1 (0.3)		0 (0)		1 (1.3)	
	Single	241 (60.4)		198 (61.7)		43 (55.1)	
	In relationship	122 (30.6)		96 (29.9)		26 (33.3)	
	Other	2 (0.5)		2 (0.6)		0 (0)	
Age (y), mean (SD; range)			20.3 (2.6; 18-35)		20.2 (2.5; 18-35)		20.4 (2.5; 18-34)
BMI (kg/m^2^), mean (SD; range)			24.6 (4.2; 16.6-35.6)		25.2 (4.2; 16.9-35.6)		22.2 (3.2; 16.6-33.2)
Percentage of body fat, mean (SD; range)			30 (8.2; 6.1-48.7)		31.1 (8; 11.4-48.7)		25.5 (7.4; 6.1-45.8)
Highest BMI (kg/m^2^), mean (SD; range)			26.2 (4.8; 17-45.0)		27.0 (4.8; 17.5-45.0)		23.1 (3.6; 17-37.9)
Highest BMI duration (d), mean (SD; range)			202 (276; 0-1825)		189 (256; 0-1460)		253 (340; 2-1825)
Days since highest BMI, mean (SD; range)			461 (642; 0-5110)		467 (649; 0-5110)		434 (614; 0-3650)
Current BMI duration (d), mean (SD; range)			237 (421; 1-5475)		191 (392; 1-5475)		429 (485; 1-2555)

**Table 2:** Characteristics of the sample at 6-month follow-up (n=221).

Characteristic		Total, n (%)	Bulimic syndrome group (n=167), n (%)	Control group (n=54), n (%)
Female		221 (100)	167 (100)	54 (100)
**Gender**				
	Woman	221 (100)	167 (100)	54 (100)
**Ethnicity**				
	Hispanic or Latino	64 (29)	50 (29.9)	14 (25.9)
	Not Hispanic or Latino	157 (71)	117 (70.1)	40 (74.1)
**Race**				
	American Indian or Alaska Native	1 (0.4)	1 (0.6)	0 (0)
	Asian	9 (4.1)	5 (3)	4 (7.4)
	Black	27 (12.2)	19 (11.4)	8 (14.8)
	Hawaiian or other Pacific Islander	1 (0.4)	1 (0.6)	0 (0)
	Multiracial	12 (5.4)	10 (6)	2 (3.7)
	White	171 (77.4)	131 (78.4)	40 (74.1)
**Educational level**				
	Part college	170 (76.9)	131 (78.4)	39 (72.2)
	Associate degree	8 (3.6)	4 (2.4)	4 (7.4)
	Bachelor degree	10 (4.5)	6 (3.6)	4 (7.4)
	Part graduate school	19 (8.6)	16 (9.6)	3 (5.6)
	Graduate degree	2 (0.9)	0 (0)	2 (3.7)
**Relationship status**				
	Married or having a partner	15 (6.8)	11 (6.6)	4 (7.4)
	Divorced or annulled	0 (0)	0 (0)	0 (0)
	Single	136 (61.5)	103 (61.7)	33 (61.1)
	In relationship	63 (28.5)	48 (28.7)	15 (27.8)
	Other	2 (0.9)	2 (1.2)	0 (0)
Age (y), mean (SD; range)		20.9 (2.8; 18-35)	20.9 (2.8; 18-33)	20.9 (2.9; 18-35)
BMI (kg/m^2^), mean (SD; range)		24.7 (4.4; 17.4-37.7)	25.4 (4.5; 17.4-37.7)	22.5 (3.3; 17.5-35.0)
Percentage of body fat, mean (SD; range)		30.1 (8.0; 11.3-48.6)	31.1 (8.2; 11.3-48.6)	27.2 (6.7; 17.7-45.9)
Highest BMI (kg/m^2^), mean (SD; range)		26.4 (5.1; 18-45.2)	27.4 (5.2; 18-45.2)	23.1 (2.9; 18.2-31.3)
Highest BMI duration (d), mean (SD; range)		196 (294; 1-1825)	162 (212; 1-1095)	302 (452; 1-1825)
Days since highest BMI, mean (SD; range)		460 (683; 0-4380)	513 (739; 0-4380)	294 (431; 0-1460)
Current BMI duration (d), mean (SD; range)		192 (298; 1-1825)	142 (190; 1-730)	351 (472; 7-1825)

Participants’ mean age did not differ between groups, and BMI was significantly higher in BN-S than control participants (t_141.92_=6.73; *P<*.001; Cohen *d*=0.85). Neither age nor BMI was associated with completing baseline or 6-month follow-up (*P* values ranged from .76 to .19, and Cohen d ranged from –0.13 to 0.03). However, older age (mean 20.6, SD 3.1 vs mean 20.0, SD 2.2 years; t_250.04_=2.12; *P=*.04; Cohen *d*=0.24) and lower BMI (mean 23.9, SD 3.9 vs mean 25.0, SD 4.4; t_350.24_=–2.53; *P=*.01; Cohen *d*=–0.25) at baseline predicted 12-month follow-up participation. WS was significantly greater in the BN-S group compared to control participants (t_157.57_=4.08; *P<*.001; Cohen *d*=0.52) and was not significantly associated with completing baseline or 6- or 12-month follow-up (*P* values ranged from .31 to .93, and Cohen *d* ranged from –0.11 to 0.01).

**Table 3:** Characteristics of the sample at 12-month follow-up (n=153).

Characteristic		Total, n (%)	Bulimic syndrome group (n=119), n (%)	Control group (n=34), n (%)
Female		153 (100)	119 (100)	34 (100)
**Gender**				
	Woman	153 (100)	119 (100)	34 (100)
**Ethnicity**				
	Hispanic or Latino	41 (26.8)	35 (29.4)	6 (17.6)
	Not Hispanic or Latino	112 (73.2)	84 (70.6)	28 (82.4)
**Race**				
	American Indian or Alaska Native	1 (0.7)	1 (0.8)	0 (0)
	Asian	6 (3.9)	3 (2.5)	3 (8.8)
	Black	16 (10.5)	11 (9.2)	5 (14.7)
	Hawaiian or other Pacific Islander	1 (0.6)	1 (0.8)	0 (0)
	Multiracial	11 (7.2)	8 (6.7)	3 (8.8)
	White	118 (77.1)	95 (79.8)	23 (67.6)
**Educational level**				
	Part college	110 (71.9)	88 (73.9)	22 (64.7)
	Associate degree	5 (3.3)	4 (3.4)	1 (2.9)
	Bachelor degree	12 (7.8)	8 (6.7)	4 (11.8)
	Part graduate school	15 (9.8)	13 (10.9)	2 (5.9)
	Graduate degree	5 (3.3)	2 (1.7)	3 (8.8)
**Relationship status**				
	Married or having a partner	14 (9.2)	13 (10.9)	1 (2.9)
	Divorced or annulled	0 (0)	0 (0)	0 (0)
	Single	76 (49.7)	60 (50.4)	16 (47)
	In relationship	57 (37.2)	42 (35.3)	15 (44.1)
	Other	0 (0)	0 (0)	0 (0)
Age (y), mean (SD; range)		21.9 (3.0; 19-35)	21.8 (2.9; 19-34)	22.5 (3.4; 20-35)
BMI (kg/m^2^), mean (SD; range)		24.4 (4.4; 16.7-39.8)	25.0 (4.6; 16.7-39.8)	22.5 (3.1; 17.6-33.4)
Percentage of body fat, mean (SD; range)		28.3 (7.9; 11-50.9)	29.3 (8.1; 11.0-50.9)	24.9 (6.1; 15.2-42.5)
Highest BMI (kg/m^2^), mean (SD; range)		25.9 (4.7; 17.7-45.0)	26.7 (4.8; 18.9-45)	23.0 (2.8; 17.7-32.6)
Highest BMI duration (d), mean (SD; range)		216 (363; 1-3650)	177 (196; 1-1095)	375 (702; 14-3650)
Days since highest BMI, mean (SD; range)		490 (648; 0-2555)	511 (649; 0-2555)	413 (651; 0-2190)
Current BMI duration (d), mean (SD; range)		230 (412; 0-3650)	171 (222; 0-1095)	467 (772; 7-3650)

### Eating Disorder and Psychiatric Diagnoses and Treatment

Table 4 presents *DSM-5* eating disorder and other psychiatric diagnoses and features at baseline. BN was the most common diagnosis, followed by OSFED-BN of low frequency or duration, with few participants meeting the criteria for current AN, BED, or their related OSFED. Diagnosis was significantly associated with completing baseline visits (*χ*^2^_3_=8.9; *P=*.03; φ=0.10), occurring in 7 (88%) out of 8 participants with AN binge-purging, 118 (77.1%) out of 153 participants with BN, 2 (50%) out of 4 participants with BED, and 99 (63.5%) out of 156 participants with OSFED diagnoses.

**Table 4:** Lifetime and current eating disorder and other psychiatric diagnoses and treatment at baseline (N=399).

								Total, n (%)					Bulimic syndrome group (n=321), n (%)			Control group (n=78), n (%)
**EDE^a^ current diagnosis**																
	AN^b^ binge-purging							—^c^					8 (2.5)			—
	BN^d^							—					153 (47.7)			—
	BED^e^							—					4 (1.2)			—
	OSFED^f^							—					156 (48.6)			—
	**OSFED subtypes^g^**															
		**Atypical AN^h^**														
			Broad						—	11 (3.4)				—		
			Narrow						—	9 (2.8)				—		
		**BN low frequency or duration**														
			Broad						—	136 (42.3)				—		
			Narrow						—	118 (36.8)				—		
		**BED low frequency or duration**														
				Broad			—					4 (1.2)			—	
				Narrow			—					3 (0.9)			—	
		**Other or unspecified**														
				Broad			—					5 (1.6)			—	
				Narrow			—					26 (8.1)			—	
**SCID-5^i^ lifetime ED^j^ diagnosis**																
	AN (κ=0.93)							—					42 (13.1)			—
	BN (κ=0.92)							—					230 (71.7)			—
	BED (κ=0.88)							—					62 (19.3)			—
	OSFED^k^ (κ=0.92)							—					159 (49.5)			—
	Atypical AN							—					14 (4.4)			—
	BN low frequency or duration							—					124 (38.6)			—
	BED low frequency or duration							—					11 (3.4)			—
	PD^l^							—					2 (0.6)			—
	NES^m^							—					0 (0)			—
	Other or unspecified							—					8 (2.5)			—
**SCID-5 diagnoses (κ)**																
	**Major depressive disorder**															
		Lifetime (κ=0.85)					183 (45.9)					175 (54.5)			8 (10)	
		Current (κ=0.74)					46 (11.5)					46 (14.3)			0 (0)	
		**Persistent depressive disorder**														
					Lifetime (κ=0.87)	118 (29.6)					113 (35.2)			5 (6)		
					Current (κ=0.94)	73 (18.3)					71 (22.1)			2 (3)		
		**Any mood disorder**														
					Lifetime (κ=0.98)	270 (67.7)					256 (79.8)			14 (18)		
					Current (κ=0.95)	121 (30.3)					118 (36.8)			3 (4)		
		**Alcohol use disorder**														
					Lifetime (κ=0.92)	149 (37.3)					139 (43.3)			10 (13)		
					Current (κ=0.81)	96 (24.1)					90 (28)			6 (8)		
		**Cannabis use disorder**														
					Lifetime (κ=0.90)	113 (28.3)					107 (33.3)			6 (8)		
					Current (κ=1.00)	74 (18.5)					71 (22.1)			3 (4)		
		**Any substance use disorder**														
					Lifetime (κ=0.90)	197 (49.4)					183 (57)			14 (18)		
					Current (κ=0.83)	136 (34.1)					128 (39.9)			8 (10)		
		**Any anxiety disorder**														
					Lifetime (κ=0.94)	213 (53.4)					203 (63.2)			10 (13)		
					Current (κ=0.97)	183 (45.9)					178 (55.5)			5 (6)		
		**Obsessive-compulsive disorder**														
					Lifetime (κ=0.82)	55 (13.8)					53 (16.5)			2 (3)		
					Current (κ=0.85)	46 (11.5)					45 (14)			1 (1)		
		**Posttraumatic stress disorder**														
					Lifetime (κ=0.91)	106 (26.6)					104 (32.4)			2 (3)		
					Current (κ=0.68)	49 (12.3)					49 (15.3)			0 (0)		
		**Any trauma-related disorder**														
					Lifetime (κ=0.98)	115 (28.8)					111 (34.6)			4 (5)		
					Current (κ=0.73)	63 (15.8)					62 (19.3)			1 (1)		
	**Suicide-related variables**															
		Lifetime ideation					177 (44.4)					167 (52)			10 (13)	
		Past week ideation					19 (4.8)					19 (5.9)			0 (0)	
		Lifetime suicide attempt					43 (10.8)					42 (13.1)			1 (1)	
	**Psychological treatment**															
		Inpatient: lifetime					23 (5.8)					21 (6.5)			2 (3)	
		Any treatment: lifetime					226 (56.6)					200 (62.3)			26 (33)	
		Current outpatient					64 (16)					57 (17.8)			7 (9)	
	**Current medications**															
		Stable SSRI^n^ over 8 wk					38 (9.5)					34 (10.6)			4 (5)	
		Hormonal contraceptive					183 (45.9)					146 (45.5)			37 (47)	

^a^EDE: Eating Disorder Examination.

^b^AN: anorexia nervosa.

^c^Not applicable.

^d^BN: bulimia nervosa.

^e^BED: binge-eating disorder.

^f^OSFED: other specified feeding or eating disorder.

^g^Guidelines presented by Keel [92] were followed for differential diagnosis of OSFED. There were no cases of current purging disorder due to the requirement of recurrent objectively large binge episodes as an inclusion criterion for participants with BN and related syndromes. In addition, 2 sets of criteria were used. Broad criteria were based on the key features that distinguish among the OSFEDs, such as significant weight loss of atypical AN, the presence of binge eating and inappropriate compensatory behaviors for BN of subthreshold duration or frequency, and the presence of binge eating and absence of recurrent inappropriate compensatory behavior for BED of subthreshold duration or frequency. Narrow criteria required the associated cognitive and affective features for each OSFED diagnosis so that narrow criteria for atypical AN indicated that all criteria for AN were met except for low weight, all criteria for BN were met except for duration or frequency of behavioral symptoms, and all criteria for BED were met except for duration or frequency of binge-eating episodes.

^h^Significant weight loss was defined as >5% reduction in BMI within 30 days or BMI <19 kg/m^2^.

^i^SCID-5: Structured Clinical Interview for Diagnostic and Statistical Manual of Mental Disorders, Fifth Edition.

^j^ED: eating disorder.

^k^Lifetime OSFED diagnoses were based on clinical interviewer assessments in the SCID-5. Given the possibility of meeting a lifetime diagnosis for >1 OSFED, no diagnostic hierarchy was used; however, parsimony was applied in making lifetime OSFED diagnoses to avoid diagnosing a prodromal phase or period of partial remission as a separate diagnosis.

^l^PD: purging disorder.

^m^NES: night eating syndrome.

^n^SSRI: selective serotonin reuptake inhibitor.

Most BN-S participants had a lifetime mood (256/321, 79.8%), substance use (183/321, 57%), or anxiety disorder (203/321, 63.2%), whereas most noneating disorder controls did not (14/78, 18%; 14/78, 18%; 10/78, 13%). All suicide indicators were more common in BN-S participants compared to controls (lifetime suicidal ideation: *χ*^2^_1_=39.1; *P<*.001; φ=0.32; past week suicidal ideation: *χ*^2^_1_=4.4; *P=*.04; φ=0.11; and lifetime suicide attempt: *χ*^2^_1_=9.1; *P=*.003; φ=0.15). No suicide attempts were reported in the week before the interview. Reflecting group differences, lifetime treatment for mental health was twice as likely in BN-S compared to control participants (*χ*^2^_1_=21.5; *P<*.001; φ=0.23).

Eating disorder diagnosis was not associated with participation at 6- or 12-month follow-up (Table 5 outlines 6-month follow-up data and Table 6 outlines 12-month follow-up data). Participants with current or lifetime obsessive-compulsive disorder at baseline were significantly less likely to participate at 12-month follow-up (*χ*^2^_1_=4.6; *P=*.03; φ=0.11 and *χ*^2^_1_=5.7; *P=*.01; φ=0.12, respectively). There were no other significant associations between completing baseline or follow-up visits and current or lifetime psychiatric diagnoses, suicide-related variables, or treatment.

**Table 5:** Current eating disorder and other psychiatric diagnoses and treatment at 6-month follow-up (n=221).

					Total, n (%)		Bulimic syndrome group (n=167), n (%)		Control group (n=54), n (%)
**EDE^a^ current diagnosis**									
	No eating disorder				72 (32.6)		19 (11.4)		53 (98)
	AN-bp^b^				5 (2.3)		5 (3)		0 (0)
	BN^c^				38 (17.2)		38 (22.8)		0 (0)
	BED^d^				2 (0.9)		2 (1.2)		0 (0)
	OSFED^e^				102 (46.2)		101 (60.5)		1 (2)
	**OSFED subtypes**								
		**Atypical AN^f^**							
			Broad	9 (4.1)		9 (5.4)		0 (0)	
			Narrow	5 (2.3)		5 (3)		0 (0)	
		**BN low frequency or duration**							
			Broad	62 (28.1)		62 (37.1)		0 (0)	
			Narrow	49 (22.2)		49 (29.3)		0 (0)	
		**BED low frequency or duration**							
			Broad	5 (2.3)		5 (3)		0 (0)	
			Narrow	2 (0.9)		2 (1.2)		0 (0)	
		**Other or unspecified**							
			Broad	26 (11.8)		25 (15)		1 (2)	
			Narrow	46 (20.8)		45 (26.9)		1 (2)	
**SCID-5^g^ current diagnoses**									
	Major depressive disorder				15 (6.8)		15 (9)		0 (0)
	Persistent depressive disorder				29 (13.1)		27 (16.2)		2 (4)
	Any mood disorder				47 (21.3)		45 (26.9)		2 (4)
	Alcohol use disorder				38 (17.2)		34 (20.4)		4 (7)
	Cannabis use disorder				27 (12.2)		24 (14.4)		3 (6)
	Any substance use disorder				58 (26.2)		52 (31.1)		6 (11)
	Any anxiety disorder				73 (33)		66 (39.5)		7 (13)
	Obsessive-compulsive disorder				23 (10.4)		22 (13.2)		1 (2)
	Posttraumatic stress disorder				20 (9)		18 (10.8)		2 (4)
	Any trauma-related disorder				23 (10.4)		21 (12.6)		2 (4)
	Suicidal ideation in the past week				9 (4.1)		9 (5.4)		0 (0)
**Current treatment and medications**									
	Outpatient				31 (14)		28 (16.8)		3 (6)
	SSRI^h^				16 (7.2)		15 (9)		1 (2)
	Hormonal contraceptives				75 (33.9)		57 (34.1)		18 (33)

^a^EDE: Eating Disorder Examination.

^b^AN-bp: anorexia nervosa binge-purging.

^c^BN: bulimia nervosa.

^d^BED: binge-eating disorder.

^e^OSFED: other specified feeding or eating disorder.

^f^AN: anorexia nervosa.

^g^SCID-5: Structured Clinical Interview for Diagnostic and Statistical Manual of Mental Disorders, Fifth Edition.

^h^SSRI: selective serotonin reuptake inhibitor.

**Table 6:** Current eating disorder and other psychiatric diagnoses and treatment at 12-month follow-up (n=153).

						Total, n (%)			Bulimic syndrome group (n=119), n (%)			Control group (n=34), n (%)
**EDE^a^ current diagnosis**												
	No eating disorder				63 (41.1)			30 (25.2)			33 (97)	
	AN-bp^b^				3 (2)			3 (2.5)			0 (0)	
	BN^c^				16 (10.5)			16 (13.4)			0 (0)	
	BED^d^				0 (0)			0 (0)			0 (0)	
	OSFED^e^				69 (45.1)			68 (57.1)			1 (3)	
	**OSFED subtypes**											
		**Atypical AN^f^**										
			Broad	3 (2)			3 (2.5)			0 (0)		
			Narrow	3 (2)			3 (2.5)			0 (0)		
		**BN low frequency or duration**										
			Broad	41 (26.8)			41 (34.5)			0 (0)		
			Narrow	31 (20.3)			31 (26.1)			0 (0)		
		**BED low frequency or duration**										
			Broad	3 (2)			3 (2.5)			0 (0)		
			Narrow	3 (2)			3 (2.5)			0 (0)		
		**Other or unspecified**										
			Broad	22 (14.4)			21 (17.6)			1 (3)		
			Narrow	32 (20.9)			31 (26.1)			1 (3)		
**SCID-5^g^ current diagnoses**												
	Major depressive disorder				10 (6.5)			10 (8.4)			0 (0)	
	Persistent depressive disorder				24 (15.7)			23 (19.3)			1 (3)	
	Any mood disorder				33 (21.6)			32 (26.9)			1 (3)	
	Alcohol use disorder				25 (16.3)			24 (20.2)			1 (3)	
	Cannabis use disorder				17 (11.1)			16 (13.4)			1 (3)	
	Any substance use disorder				39 (25.5)			38 (31.9)			1 (3)	
	Any anxiety disorder				44 (28.8)			43 (36.1)			1 (3)	
	Obsessive-compulsive disorder				15 (9.8)			14 (11.8)			1 (3)	
	Posttraumatic stress disorder				11 (7.2)			11 (9.2)			0 (0)	
	Any trauma-related disorder				15 (9.8)			15 (12.6)			0 (0)	
Suicidal ideation in the past week						2 (1.3)			2 (1.7)			0 (0)
**Current treatment and medications**												
	Outpatient				27 (17.6)			26 (21.8)			1 (3)	
	SSRI^h^				4 (2.6)			4 (3.4)			0 (0)	
	Hormonal contraceptives				57 (37.3)			43 (36.1)			14 (41)	

^a^EDE: Eating Disorder Examination.

^b^AN-bp: anorexia nervosa binge-purging.

^c^BN: bulimia nervosa.

^d^BED: binge-eating disorder.

^e^OSFED: other specified feeding or eating disorder.

^f^AN: anorexia nervosa.

^g^SCID-5: Structured Clinical Interview for Diagnostic and Statistical Manual of Mental Disorders, Fifth Edition.

^h^SSRI: selective serotonin reuptake inhibitor.

### Psychometric Properties of Measures

IRR was excellent for all lifetime and substantial for current SCID-5 diagnoses [119,120]. Table 4 includes κ statistics for lifetime and current SCID-5 diagnoses based on IRR assessment from an independent review of 127 interviews across the full project, including baseline and follow-up assessments. Estimates include in-person and remote interviews.

Table 7 presents internal consistency and Table 8 provides test-retest reliability for EDE interview and questionnaire scales. Cronbach α exceeded thresholds for acceptability across all assessments and compared favorably to published estimates for all measures. Test-retest reliability was good and generally higher over shorter intervals, likely reflecting true change in constructs over time.

**Table 7:** Internal consistency reliability of measures at baseline and follow-up.

Measures				Cronbach α at baseline		Cronbach α at 6 mo		Cronbach α at 12 mo
**Eating pathology**								
	**Eating Disorder Examination interview**							
		Global	0.90		0.91		0.90	
		Restraint	0.83		0.81		0.81	
		Eating concern	0.76		0.77		0.81	
		Shape concern	0.92		0.91		0.91	
		Weight concern	0.86		0.87		0.82	
	**Self-report**							
		Clinical impairment assessment	0.96		0.97		0.96	
		Body shape questionnaire	0.98		0.99		0.98	
	**Three-Factor Eating Questionnaire**							
		Cognitive restraint	0.92		0.93		0.91	
		Disinhibition	0.90		0.89		0.90	
		Hunger	0.78		0.79		0.74	
**Reward or inhibition**								
	Behavioral inhibition scale		0.81		0.77		0.79	
	**Behavioral Activation Scale**		0.85		0.89		0.88	
		Drive	0.78		0.84		0.86	
		Fun seeking	0.73		0.74		0.79	
		Reward responsiveness	0.73		0.77		0.77	
	**Sensitivity to Reward and Sensitivity to Punishment Questionnaire**							
		Sensitivity to reward	0.77		0.78		0.81	
		Sensitivity to punishment	0.89		0.88		0.89	
**Affect**								
	**Positive and Negative Affect Schedule**							
		Positive affect	0.89		0.90		0.94	
		Negative affect	0.89		0.92		0.93	

**Table 8:** Test-retest reliability of measures across assessment waves.

Measures				Baseline to 6 mo, *r*		6 to 12 mo, *r*		Baseline to 12 mo, *r*
**Eating pathology**								
	**Eating Disorder Examination interview**							
		Global	0.85		0.79		0.76	
		Restraint	0.74		0.64		0.67	
		Eating concern	0.65		0.60		0.53	
		Shape concern	0.84		0.79		0.76	
		Weight concern	0.79		0.78		0.70	
	**Self-report**							
		Clinical impairment assessment	0.79		0.82		0.71	
		Body shape questionnaire	0.85		0.85		0.82	
	**Three-Factor Eating Questionnaire**							
		Cognitive restraint	0.85		0.82		0.82	
		Disinhibition	0.84		0.78		0.78	
		Hunger	0.70		0.59		0.60	
**Reward or inhibition**								
	Behavioral Inhibition Scale		0.68		0.78		0.70	
	**Behavioral Activation Scale**		0.68		0.68		0.57	
		Drive	0.65		0.57		0.54	
		Fun seeking	0.65		0.68		0.58	
		Reward responsiveness	0.59		0.65		0.50	
	**Sensitivity to Reward and Sensitivity to Punishment Questionnaire**							
		Sensitivity to reward	0.68		0.75		0.66	
		Sensitivity to punishment	0.83		0.90		0.82	
**Affect**								
	**Positive and Negative Affect Schedule**							
		Positive affect	0.59		0.79		0.67	
		Negative affect	0.64		0.65		0.49	

## Discussion

### Principal Findings

The study protocol is designed to test an explanatory biobehavioral model for the association between WS and severity and maintenance of BN and related eating disorders characterized by binge eating. For BN-S severity, we expect significant cross-sectional associations between greater WS, lower leptin levels, lower GLP-1 response, greater reward valuation, and lower reward satiation. We also anticipate that greater reward valuation will be significantly associated with higher LOC frequency and a significant indirect pathway from greater WS to higher LOC frequency via lower leptin levels, lower GLP-1 response, and greater reward valuation. Furthermore, we expect that lower reward satiation will be significantly associated with larger eating/binge-eating episode size and a significant indirect pathway from greater WS to larger eating/binge-eating episode size via lower leptin levels, lower GLP-1 response, and lower reward satiation. If supported, findings would demonstrate that biological concomitants of WS explain variance in binge-eating severity via alterations in reward valuation and reward satiation. For BN-S maintenance (vs remission), we hypothesized significant prospective associations between these variables, with reward valuation and reward satiation temporally mediating associations between WS as the exposure and changes in LOC frequency and eating/binge-eating episode size, respectively, as the outcomes. If supported, findings would have implications for the assessment, diagnosis, and future clinical trials of eating disorders characterized by binge eating.

Current standardized eating disorder assessments, including the SCID-5 [93] and the EDE 17.0D [83], secure information about current body weight and lowest body weight in relation to height. However, neither includes a question about the highest lifetime adult weight. If findings support hypotheses, then future assessments would benefit from including this question to calculate WS as a prognostic indicator.

Results may inform diagnostic criteria for eating disorders. The *DSM-5* currently uses BMI as a severity indicator for AN, frequency of inappropriate compensatory behaviors for BN, and frequency of binge-eating episodes for BED [5]. If our model supports WS as a marker of severity transdiagnostically, then WS could provide a common metric across eating disorders and potentially explain differences in outcomes among them. Beyond broad, transdiagnostic implications, findings may also refine the discrepancies between the *DSM-5* and *International Classification of Diseases, 11th Revision* (*ICD-11*) in the definition of a binge-eating episode for diagnosis of BN and BED. Our model focuses on the *DSM-5* definition, which requires the consumption of an objectively large amount of food in addition to LOC. The *ICD-11* requires a subjective LOC combined with eating either “notably more or differently than usual” [121]. If findings support posited pathways from WS to episode size and LOC, this would support the validity of the *DSM-5* definition over the *ICD-11* definition.

Finally, given recent interest and controversy surrounding the use of GLP-1 agonists for binge eating (eg, semaglutide in Wegovy and Ozempic) [122,123], these longitudinal data, funded solely by the NIMH and the US Department of Education, can elucidate mixed findings from early clinical trials [124-126] and inform the design of future investigations. Specifically, our model contextualizes the impact of GLP-1 function for those who have lost weight, predicting greater disruptions associated with greater WS. Previous studies supporting the potential efficacy of GLP-1 agonists have relied on secondary analyses of data collected in open trials [125-127] or a randomized controlled treatment trial for obesity without placebo control [128]. In the sole double-blind, randomized controlled trial testing a GLP-1 agonist against a placebo for the treatment of BED [124], no significant differences emerged in remission of binge eating or BED. Similar to other studies of GLP-1 agonists, weight loss was significantly greater in the active compared to the control condition. However, participants on placebo lost only 0.9 (SD 0.7) kg over 17 weeks. Comparing improvements in binge eating on a placebo without weight loss may not provide a valid comparison against those treated with liraglutide, who lost 4.7 (0.8) kg of weight and experienced decreases in binge eating [124]. If our hypotheses are supported, future evaluations of GLP-1 efficacy will examine WS at intake and relative changes in weight over treatment as covariates in predicting changes in binge-eating severity.

Strengths of the project include the large, diverse sample; inclusion of multiple units of analysis; and longitudinal design, with high retention across multiple visits at baseline and high retention over follow-up. We retained 290 (72.7%) out of 399 women for all clinical, behavioral, and biological assessments at baseline, and 249 (85.8%) of these 290 women provided longitudinal data. This supports the feasibility of our approach even in the face of unanticipated challenges encountered with the onset of the COVID-19 pandemic. Studies using laboratory-based feeding paradigms [81] have included anywhere from 7 to 103 participants in PR tasks and fixed or ad-lib meals. Our combination of all methods in 290 participants far exceeds these benchmarks. Furthermore, most biobehavioral studies were constrained by their cross-sectional design, limiting conclusions regarding the role of observed disruptions as correlates, consequences, or contributors to pathology. Our measures had strong psychometric properties that minimized random error, and initial analyses indicate that effect sizes will exceed original estimates. Thus, we will have sufficient power for prospective analyses.

Project weaknesses include the absence of sexual orientation information and the exclusion of male participants. Eating disorders, including AN binge-purging, BN, BED, and their OSFED variants, predominantly affect female individuals [129,130]. Although restricting recruitment to women limits generalizability to men due to potential biologically based differences in the influence of ovarian hormones on GLP-1 function [65], it was not feasible to recruit enough men to directly examine sex as a biological variable. This limitation was due to the large sample size required for adequately powered analyses and the low prevalence of eating disorders in men. Despite this limitation, findings may impact the assessment, diagnosis, and treatment of a majority of those with eating disorders characterized by binge eating, given the preponderance of these disorders in women [129,130]. Furthermore, the sample demonstrated limited diversity in socioeconomic status. Participation requirements were likely too high for most individuals with limited resources or full-time commitments at work or home. These factors will impact the generalizability of findings. Attrition was related to age, BMI, and the presence of a BN-S at baseline. Greater attrition in BN-S may reflect a higher participant burden in this group, including longer interview duration due to greater pathology and behavioral task duration due to higher RVE and lower reward satiation. BN-S participants endorsed greater impairment, which may extend to impaired ability to follow through with study participation. Among participants who provided full baseline data and were eligible for longitudinal follow-up, we did not observe significant differences between BN-S and control groups. We advise using all available data with imputation for missing values to minimize the influence of biased attrition.

Finally, in using the RDoC approach to participant recruitment, we did not set a priori goals for the recruitment of participants who would fall into specific *DSM-5* diagnostic groups, and this may have limited the extent to which our sample has a transdiagnostic representation of all eating disorders characterized by binge eating. The requirement that participants be medically healthy and free of medications that could influence weight or appetite was necessitated by our interest in biological factors underlying RVE and satiation, consistent with the RDoC approach. This may have restricted the number of participants who presented with AN or BED, given the associations between extreme BMI and medical conditions. This, combined with our age range, may explain the large number of participants with *DSM-5* BN and OSFED characterized as BN with low duration or frequency. The latter group could lead to misinterpretation of the sample as including participants with “subthreshold” eating disorders. However, all participants had a full-threshold *DSM-5* eating disorder. Further, the minimum behavioral symptom frequency ensured that all participants met the minimum symptom frequency required for a *DSM-5* diagnosis of BED. Many of our OSFED participants were engaging in binge eating frequently enough for a diagnosis of BN or BED, but the frequency of inappropriate compensatory behaviors was too high for a diagnosis of BED and too low for a diagnosis of BN.

### Conclusions

Preliminary data from subsets of participants in this project have been included in other reports [1,79,131-133]; however, this is the first report presenting data from all participants and all waves. Future papers will focus on testing our model to predict the severity and maintenance of BN-S. Beyond testing our RDoC-informed model, biological and behavioral variables will be examined in novel combinations as predictors of changes in eating disorder expression and comorbidity. A careful review of this paper will help researchers accessing data through the NDA identify which data are most relevant (eg, active vs total GLP-1), their context (eg, pre– vs post–COVID-19 pandemic), and other factors that may influence interpretations. Grounding analyses with a full understanding of methods will facilitate rigorous and reproducible research.

## Appendix

Multimedia Appendix 1 Peer-review report from the Center for Scientific Review Special Emphasis Panel Member Conflict: Sleep, Memory, Anxiety, and Reward (ZRG1 BBBP-Z [02]; National Institutes of Health).

## Abbreviations

AN: anorexia nervosa
Arc: arcuate nucleus
BED: binge-eating disorder
BN: bulimia nervosa
BN-S: bulimia nervosa and related syndromes
CART: cocaine- and amphetamine-regulated transcript
CV: coefficient of variation
DSM-5: Diagnostic and Statistical Manual of Mental Disorders, Fifth Edition
EDE: Eating Disorder Examination
FSU: Florida State University
GLP-1: glucagon-like peptide 1
HIPAA: Health Insurance Portability and Accountability Act
ICC: intraclass correlation
ICD-11: International Classification of Diseases, 11th Revision
IRB: institutional review board
IRR: interrater reliability
LOC: loss of control
NAc: nucleus accumbens
NDA: National Institute of Mental Health Data Archive
NIMH: National Institute of Mental Health
NPY/AgRP: neuropeptide Y and agouti-related protein
NTS: nucleus of the solitary tract
OSFED: other specified feeding or eating disorder
PR: progressive ratio
RDoC: Research Domain Criteria
RVE: reward valuation effort
SCID-5: Structured Clinical Interview for Diagnostic and Statistical Manual of Mental Disorders, Fifth Edition
SEM: structural equation model
VAS: Visual Analog Scale
VTA: ventral tegmental area
WS: weight suppression

## Data Availability

The datasets generated or analyzed during this study are available in the National Institute of Mental Health Data Archive repository [134].
