# Supplementary material for: Examining Weight Suppression, Leptin Levels, Glucagon-Like Peptide 1 Response, and Reward-Related Constructs in Severity and Maintenance of Bulimic Syndromes: Protocol and Sample Characteristics for a Cross-Sectional and Longitudinal Study
Source: JMIR Res Protoc. 2025 Apr 8;14:e66554. doi: 10.2196/66554 (PMC12015349; doi:10.2196/66554)
Supplement: Multimedia Appendix 1 [file resprot_v14i1e66554_app1.pdf]

SUMMARY STATEMENT
(Privileged Communication)

PROGRAM CONTACT:
Mark Chavez
301-443-8942
mchavez1@mail.nih.gov

Release Date: 03/28/2016 12:35 PM
Revised Date:

Application Number: 1 R01 MH111263-01

Principal Investigator

KEEL, PAMELA K.

Applicant Organization: FLORIDA STATE UNIVERSITY

Review Group: ZRG1 BBBP-Z (02)
Center for Scientific Review Special Emphasis Panel
Member Conflict: Sleep, Memory, Anxiety and Reward

Meeting Date: 03/07/2016
Council: MAY 2016
Requested Start: 07/01/2016
RFA/PA: PA13-302
PCC: A2-AIM
Dual PCC: BN/SVA
Dual IC(s): DA, DK

Project Title: Biobehavioral Prediction of Illness Trajectory in Bulimic Syndromes

SRG Action: Impact Score:30 Percentile:16 #
Next Steps: Visit http://grants.nih.gov/grants/next_steps.htm
Human Subjects: 30-Human subjects involved - Certified, no SRG concerns
Animal Subjects: 10-No live vertebrate animals involved for competing appl.
Gender: 1A-Both genders, scientifically acceptable
Minority: 1A-Minorities and non-minorities, scientifically acceptable
Children: 1A-Both Children and Adults, scientifically acceptable
Clinical Research - not NIH-defined Phase III Trial

Project Year	Direct Costs Requested	Estimated Total Cost
1	291,128	432,154
2	334,457	496,473
3	338,548	502,545
4	341,044	506,251
5	319,074	473,638
TOTAL	1,624,251	2,411,061

ADMINISTRATIVE BUDGET NOTE: The budget shown is the requested budget and has not been adjusted to reflect any recommendations made by reviewers. If an award is planned, the costs will be calculated by Institute grants management staff based on the recommendations outlined below in the COMMITTEE BUDGET RECOMMENDATIONS section.

1R01MH111263-01 Keel, Pamela

RESUME AND SUMMARY OF DISCUSSION: This application proposes to test a model of bulimic syndromes (BN-S) that causally links severity to weight suppression via reduced leptin levels, blunted response to postprandial glucagon-like peptide 1 (GLP-1), and changes in approach motivation and reward system responsiveness. This is an ambitious project that addresses an important topic; linking biomarkers to behavioral measures of motivation and reward responsivity is significant, and results from this project could result in development of more effective treatments for binge eating. This project is well-articulated and presents good preliminary data. However, the committee had some concerns regarding the approach. There were concerns that the role of leptin in the model is somewhat oversimplified, the role of body mass index (BMI) in the model is not entirely clear, and the proposed sample size may not be feasible. Overall, this is an ambitious project on a significant topic from an excellent investigator with some weaknesses in the approach that detract somewhat from its high potential impact.

DESCRIPTION (provided by applicant): Bulimic syndromes (BN-S) are characterized by large out-of-control binge episodes, span the three DSM-5 eating disorder categories of anorexia nervosa-binge purge subtype (ANbp), bulimia nervosa (BN), and binge eating disorder (BED), and vary considerably in illness severity and course. Our long-term objectives are to identify biobehavioral predictors of illness trajectory in BN-S so that treatments may be developed to address key factors influencing the severity and course of binge eating. The specific aims of this study are to test a model in which weight suppression (WS) leads to deficient circulating leptin, which contributes to blunted postprandial glucagon-like peptide 1 (GLP-1) response, which causes alterations in the RDoC core constructs of approach motivation and sustained responsiveness to reward, which then contribute to BN-S severity and maintenance. WS (the difference between an individual's highest weight and current weight) has emerged as one key predictor of severity and maintenance of BN-S, and investigators have posited a biobehavioral mechanism for this association. Yet, no study has evaluated how the biological consequences of WS may contribute to alterations in RDoC core constructs proposed to contribute to binge eating. Our model posits that the same set of physiological consequences of WS contribute to binge eating by 1) increasing drive/motivation to consume food (approach motivation), and 2) decreasing ability for food consumption to lead to a state of satiation/satisfaction (sustained responsiveness to reward). Approach motivation will be measured both behaviorally as breakpoint on progressive ratio tasks for food and non-food reinforcers and by self-report. Sustained responsiveness to reward (satiation) will be measured both behaviorally as food intake in an ad lib meal and by self-report. Participants (N=320) with BN-S and non-eating disorder controls, ranging in BMI from 16 to 35 kg/m2, will be assessed for WS, leptin, GLP-1 response to a fixed meal, approach motivation, ability to achieve satiation in an ad lib meal, self-report measures of core constructs, and binge eating at baseline, 6-, and 12-month follow-up, to produce the first study of biobehavioral predictors of illness trajectory in BN-S transdiagnostically. Examining the integration of approach motivation and satiation through the same set of physiological mechanisms represents a major innovation as it translates cutting-edge research in basic science to understand clinical phenomena in BN-S. If differences in illness trajectory across ANbp, BN, and BED are attributable to the underlying dimension of WS, this will fundamentally alter conceptualization of BN-S from three eating disorder categories to one eating disorder. If findings support biobehavioral distinctions across the BMI range, this would provide important information in what treatments would work in whom. Moreover, our focus on factors that are modifiable via behavioral and pharmacological interventions increases the public health significance of this work by facilitating novel treatment approaches to ameliorate distress, disability, and death in syndromes accounting for an important segment of those suffering from eating disorders.

PUBLIC HEALTH RELEVANCE: Bulimic syndromes impact over 5% of the US population, represent a leading cause of disability in young women, and are associated with increased risk of death, particularly death by suicide. The proposed study seeks to identify mechanisms that contribute to severity and maintenance of illness. Findings will contribute to enhanced conceptualization, assessment, and treatment of these deadly diseases.

CRITIQUE 1:

Significance: 2
Investigator(s): 1
Innovation: 2
Approach: 4
Environment: 2

Overall Impact: This application proposes an ambitious project to test hypotheses regarding weight suppression, biomarkers (leptin, GLP-1), RDoC positive valence constructs, and the severity and maintenance of binge eating. The sample will include 260 symptomatic women across a wide range of BMIs and 60 control subjects. The primary indices of severity are the size of binge episodes and the degree of loss of control. The Principal Investigator is experienced and highly regarded, the application is tightly reasoned, and promising preliminary results are described. There has been substantial recent interest in the importance of weight suppression in impacting the course and outcome of eating disorders, especially of Bulimia Nervosa (BN), and this application would examine the effect of weight suppression across a much broader diagnostic range, consistent with RDoC. Concerns regarding the model and the conclusions that could be drawn from the work proposed mitigate these strengths.

1. Significance:

Strengths

- Binge eating is a relatively common behavioral problem, and is associated with significant psychological and physical morbidity. The biobehavioral mechanisms that underlie binge eating are poorly understood. The successful identification of biomarkers significantly related to severity and to course of symptoms should facilitate the development of more effective treatment strategies.
- The ability to link biomarkers to behavioral measures of motivation and reward responsivity would considerably advance the field, by explicitly connecting biological and behavioral parameters.

Weaknesses

- The application aims to assess associations between biological factors, behavioral measures, and course of symptoms, but will not rigorously assess the causative model proposed. This may limit the direct application of the results of this study to understanding of causal links and to treatment development.

2. Investigator(s):

Strengths

- The Principal Investigator is a prominent figure in the eating disorders field, and is highly regarded for the quality of her prior research work and for the clarity of her thinking.
- Her colleagues, with whom the Principal Investigator has previously worked, provide necessary expertise in the biology of leptin and GLP-1 and their assays, blood sampling via intravenous catheters, and statistics.

Weaknesses

- None noted.

3. Innovation:

Strengths

- The application is innovative in examining the associations between a range of parameters, including body weight, plasma hormones, behavioral assessments of motivation and of reward, and clinical course. Each of these has been examined separately, but it is a significant innovation to assess them all over time in a large number of subjects.

Weaknesses

- No major weaknesses noted.

4. Approach:

Strengths

- A major strength of the application is the articulation of a specific biobehavioral model that incorporates clinical, biological, and behavioral elements.
- The use of objective behavioral tasks to assess motivation and reward (satiation) is novel and a significant strength.
- Preliminary studies support a number of the elements of the proposed model.

Weaknesses

- Most, though not all, of the results that provide the basis for the proposed model are from studies of Bulimia Nervosa, and are only partially supportive. For example, prior work found no association between leptin and binge frequency or binge size in a study of 32 patients with Bulimia Nervosa, and only a marginal correlation between weight suppression and leptin (Bodell & Keel, 2015). Naessen et al (2011) reported reduced post-prandial GLP-1 in Bulimia Nervosa, but no apparent relationship to symptoms.
- Studies of other populations, such as Anorexia Nervosa and broadly defined eating disorders, have failed to document a strong association between weight suppression and binge eating. Therefore, there is a concern that the impact of weight suppression across the weight spectrum may not be as great as hypothesized.
- A key part of the hypothesis is that leptin is an index of weight suppression. It is well established that the primary determinant of leptin concentration is body fat. A potential problem is that, even after weight loss, a strong relationship between BMI and leptin will remain, obscuring the impact of weight suppression *per se*. Although some of the preliminary data suggest a relationship between weight suppression and leptin among

individuals with Bulimia Nervosa, it is not clear that an identical relationship will hold true over the much larger BMI range to be examined.

- The model posits that the effect of leptin on severity occurs via an impact on the post-prandial release of GLP-1. Given the multiple CNS effects of leptin, it seems that more direct effects of leptin should also be considered.
- This application will examine the association between parameters posited to be causally linked, but, as noted in the application, the project will not rigorously test the causal links, for example, by a pharmacological manipulation. The argument that the proposed study is a necessary first step is not entirely convincing.

5. Environment:

Strengths

- The environment is excellent. The investigators have successfully conducted similar studies in the past, and the preliminary data are supportive of their ability to carry out the work proposed.

Weaknesses

- No major weaknesses noted.

Protections for Human Subjects:

Acceptable Risks and/or Adequate Protections

- The proposed studies are of minimal risk.

Data and Safety Monitoring Plan (Applicable for Clinical Trials Only):

Not Applicable (No Clinical Trials)

Inclusion of Women, Minorities and Children:

- Sex/Gender: Distribution justified scientifically
- Race/Ethnicity: Distribution justified scientifically
- Inclusion/Exclusion of Children under 21: Including ages < 21 justified scientifically
- Only women, ages 18-35 years, will be included. Given the distribution of eating disorders in the population, this is acceptable. The racial/ethnic distribution appears appropriate.

Vertebrate Animals:

Not Applicable (No Vertebrate Animals)

Biohazards:

Not Applicable (No Biohazards)

Budget and Period of Support:

Recommend as Requested

CRITIQUE 2:

Significance: 1
Investigator(s): 1
Innovation: 2
Approach: 4
Environment: 1

Overall Impact: Accumulating evidence over the past 10 years has identified weight suppression as a reliable predictor of a wide variety of eating disorder characteristics in those with both anorexia (AN) and bulimia nervosa (BN). Furthermore, weight suppression is a risk factor that overlaps little with previously studied risk factors for eating disorders (e.g., overvaluation of weight and shape, personality predispositions, dietary restraint) and therefore has substantial potential to supplement existing literature on risk factors for disordered eating. The application delineates a well-reasoned and very well-supported model connecting weight suppression, leptin, GLP-1 with reward- and satiation-based vulnerabilities that perpetuate binge eating. The pilot data systematically support all links in the proposed model. Therefore the potential of the proposed study to contribute novel findings on risk factors for bulimic disorders is very high. Nonetheless, there are number of unanswered questions and weaknesses that somewhat detract from the strengths of this application.

1. Significance:

Strengths

- Although accumulating evidence indicates that weight suppression is a cross-sectional and prospective predictor of various eating disorder characteristics in anorexia and bulimia nervosa, little is known about the mechanism(s) responsible for its predictive effects. This application holds great promise in elucidating both biological and behavioral mechanisms affecting bulimic spectrum eating disorders.
- The application provides compelling research and pilot data that make the multivariate model of the connection between weight suppression, hormonal mediators, binge eating and eating disorder duration and severity explicit, plausible and testable. If even only some of the hypotheses are supported, the results could inform the development of new pharmacological and behavioral interventions.
- The application is well integrated with the RDoC's model, based on both its transdiagnostic perspective and its explicit delineation of dimensions specified in RDoCs.

Weaknesses

- In the model, factors driving over-consumption are repeatedly emphasized, but the possible roles of inhibitory factors also affecting intake (dietary restraint, possible early satiety in emaciated participants) are barely mentioned. Because binge eating disorders usually involve a constant battle between approach (reward) and avoidance (restraint), there is insufficient consideration of this inhibitory side of the equation and how it will be accounted for.

- In literature on weight suppression in eating disorders, there are several studies indicating that current BMI affects eating disorder characteristics along with, or in interaction with, weight suppression (WS). This might especially be the case in the proposed research since BMIs will range from the mid-teens to the mid-30s. The application does not sufficiently discuss the possible influence of BMI, alone and in conjunction with WS, on the measures and outcomes.

2. Investigator(s):

Strengths

- Principal Investigator Keel and Co-investigator Williams are very well qualified, in terms of both expertise and relevant past experience and accomplishments, to undertake the proposed research. Dr. Keel is a highly accomplished researcher in eating disorders and Dr. Williams is a leading researcher on how interactions between the central nervous system (CNS) and peripheral hormones impact food reward. Consultant Crosby is also first rate for statistical guidance.

Weaknesses

- None noted.

3. Innovation:

Strengths

- Weight suppression is not novel but it is largely independent of most known risk factors for eating disorders, so this in-depth study is likely to yield a number of novel findings.
- The vast majority of eating disorders research has examined predictors of course within those with AN, BN or binge eating discourse (BED); the current application's focus on any eating disorder involving recurrent binge eating, across a broad BMI spectrum, is innovative.
- Although some past studies have examined the consequences of weight suppression over time, the proposed study does so as part of a well-reasoned and well-supported biobehavioral model that will also permit mediational tests of the proposed model.
- The thorough integration of animal data relating GLP-1 to reward and satiation with human data on weight loss, leptin and GLP-1 was innovative.

Weaknesses

- No major weaknesses noted.

4. Approach:

Strengths

- There are many positive features of the approach including a large sample size, a long-term prospective design, and inclusion of a non-disordered control group, tests of mediation and the inclusion of a variety of behavioral and biological predictive measures.

Weaknesses

- The application lists eating disorder "severity" as a primary dependent measure but it is not clear how severity is being defined. Binge size is mentioned, though this is not usually even measured in eating disorder studies. Binge eating and purging frequency are presumably relevant but are not discussed as part of eating disorder severity.
- The same weight loss in an anorexic and obese individual may have quite different biological and behavioral consequences, but this is not adequately addressed.
- Many studies have found that weight suppression predicts future weight change. However, these findings and their implications for the project's hypotheses receive insufficient attention. In addition, if higher weight suppression predicts greater weight gain at 6 and 12 month follow-ups, the greater weight gain among those higher in weight suppression would presumably lessen the severity of the eating disorder at these time points. The application does not sufficiently address this possibility.
- There are many peptides and hormones that are affected by weight loss and that affect food intake and body weight. The focus on leptin and GLP-1 is not clearly, entirely motivated.
- Some past research involving lab-based food intake in binge eaters has allowed participants to purge via vomiting if they chose to do so. The application does not discuss the possibility that food intake could be reduced if the bulimic participants do not have the ability to purge after eating.
- The project will be recruiting binge eating participants who fall into AN, BN or BED categories; nonetheless their proposed sample size of 260 appears to be unrealistically large. The feasibility of this is unclear. Furthermore, it appears that a high proportion of participants may be college students who would likely have disorders of lesser severity and duration. This could restrict the range on some of the measures and reduce the chances of obtaining significant results.
- It is unclear how the project takes into account the possibility that some participants may have never played the Angry Birds game or will not find it motivating.
- There are some inconsistencies and weaknesses in the weight suppression literature that are not well addressed (e.g., prediction of therapy outcome; inconsistent relationships with binge eating).

5. Environment:

Strengths

- The research environment at Florida State University and the labs of the investigators are fully prepared to carry out the proposed research.

Weaknesses

- None noted.

Protections for Human Subjects:

Acceptable Risks and/or Adequate Protections

Data and Safety Monitoring Plan (Applicable for Clinical Trials Only):

Acceptable

Inclusion of Women, Minorities and Children:

- Sex/Gender: Distribution justified scientifically
- Race/Ethnicity: Distribution justified scientifically
- Inclusion/Exclusion of Children under 21: Including ages < 21 justified scientifically

Vertebrate Animals:

Not Applicable (No Vertebrate Animals)

Biohazards:

Not Applicable (No Biohazards)

Budget and Period of Support:

Recommend as Requested

- All of Dr. Keel's and Dr. Williams' efforts during the academic year appear to be cost shared in their entirety.

Additional Comments to Applicant:

- The use of an index that examines weight suppression in relation to highest past weight, in addition to raw weight suppression scores, could address the issue of different biological and behavioral consequences for weight loss in anorexic and obese individuals.

CRITIQUE 3:

Significance: 3
Investigator(s): 1
Innovation: 2
Approach: 3
Environment: 1

Overall Impact: The proposed study from a Principal Investigator who is a leader in eating disorders research would provide an innovative, rigorous, sophisticated approach to understanding the pathophysiology, correlates, and course, of bulimia syndromes (BN-S) in a wide range of women exhibiting BN-S characteristics. The study has the potential to inform both conceptual and empirical approaches to eating disorders and is innovative in its focus on reward systems, inclusion of multiple measures of each construct, longitudinal design, and clear conceptual model for interplay of behavior, hormones, and clinical characteristics. However, the model for the proposed study represents a somewhat oversimplified view of the role of leptin and the numerous factors that contribute to obesity and BN symptoms. In particular, the complex role of leptin in satiety, eating behavior, and weight is not captured adequately. In the design, the rationale for including only women is not compelling. This becomes an issue at the binge-eating (but not purging) end of the BN-S spectrum, where prevalence is closer to even

between men women than in the bingeing-and-purging subpopulation, which is mostly female. Potential covariates (e.g., age, clinical characteristics, SSRIs) are not addressed adequately. This is particularly concerning for BMI, which is more strongly related to BN symptoms for the binge-eating-only subsample than for the also-purging subsample. In all, this application addresses a high-impact question of both clinical and scientific relevance, reflects a strong Principal Investigator and environment, uses very strong methods, and has several small weaknesses.

Protections for Human Subjects:

Acceptable Risks and/or Adequate Protections

- Risks are adequately identified and addressed.

Data and Safety Monitoring Plan (Applicable for Clinical Trials Only):

Acceptable

Inclusion of Women, Minorities and Children:

- Sex/Gender: Distribution justified scientifically
- Race/Ethnicity: Distribution justified scientifically
- Inclusion/Exclusion of Children under 21: Including ages < 21 justified scientifically
- The proposed study will include female participants as young as 18 years, based on the prevalence of BN-S. The sample will be diverse, and the proposed recruitment includes a plan to enhance participation of women from minority populations.

Vertebrate Animals:

Not Applicable (No Vertebrate Animals)

Biohazards:

Not Applicable (No Biohazards)

Budget and Period of Support:

Recommend as Requested

THE FOLLOWING SECTIONS WERE PREPARED BY THE SCIENTIFIC REVIEW OFFICER TO SUMMARIZE THE OUTCOME OF DISCUSSIONS OF THE REVIEW COMMITTEE, OR REVIEWERS' WRITTEN CRITIQUES, ON THE FOLLOWING ISSUES:

PROTECTION OF HUMAN SUBJECTS (Resume): ACCEPTABLE

INCLUSION OF WOMEN PLAN (Resume): ACCEPTABLE

INCLUSION OF MINORITIES PLAN (Resume): ACCEPTABLE

INCLUSION OF CHILDREN PLAN (Resume): ACCEPTABLE

COMMITTEE BUDGET RECOMMENDATIONS: The budget was recommended as requested.

Footnotes for 1 R01 MH111263-01; PI Name: Keel, Pamela K.

Ad hoc or special section application percentiled against "Total CSR" base.

NIH has modified its policy regarding the receipt of resubmissions (amended applications). See Guide Notice NOT-OD-14-074 at http://grants.nih.gov/grants/guide/notice-files/NOT-OD-14-074.html. The impact/priority score is calculated after discussion of an application by averaging the overall scores (1-9) given by all voting reviewers on the committee and multiplying by 10. The criterion scores are submitted prior to the meeting by the individual reviewers assigned to an application, and are not discussed specifically at the review meeting or calculated into the overall impact score. Some applications also receive a percentile ranking. For details on the review process, see http://grants.nih.gov/grants/peer_review_process.htm#scoring.

MEETING ROSTER

The roster for this review meeting is displayed as an aggregated roster that includes reviewers from multiple CSR Special Emphasis Panels of the Biobehavioral and Behavioral Processes Integrated Review Group
for the 2016/05 council round.
This roster for CSR is available at:
http://public.era.nih.gov/pubroster/Reports?DOCTYPE=SEP&DESFORMAT=PDF&AGENDA_SEQ_NUM_P=305287
